# The Wnt Signaling Antagonist Kremen1 is Required for Development of Thymic Architecture 

**DOI:** 10.1080/17402520600935097

**Published:** 2006

**Authors:** Masako Osada, Emi Ito, Hector A. Fermin, Edwin Vazquez-Cintron, Tadmiri Venkatesh, Roland H. Friedel, Mark Pezzano

**Affiliations:** 1 Department of Biology The City College of the City University of New York RCMI Center for the Study of the Cellular and Molecular Basis of Development 138th Street and Convent Avenue New York NY 10031 USA; 2 Department of Biological Sciences Stanford University 371 Serra Mall Stanford CA 94305 USA

## Abstract

Wnt signaling has been reported to regulate thymocyte proliferation and selection at several stages during T cell ontogeny, as well as the expression of *FoxN1* in thymic epithelial cells (TECs). Kremen1 (Krm1) is a negative regulator of the canonical Wnt signaling pathway, and functions together with the secreted Wnt inhibitor Dickkopf (Dkk) by competing for the lipoprotein receptor-related protein (LRP)-6 co-receptor for Wnts. Here *krm1* knockout mice were used to examine *krm1* expression in the thymus and its function in thymocyte and TEC development. *krm1* expression was detected in both cortical and medullary TEC subsets, as well as in immature thymocyte subsets, beginning at the CD25+CD44+ (DN2) stage and continuing until the CD4+CD8+(DP) stage. Neonatal mice show elevated expression of *krm1* in all TEC subsets. *krm1*^− / −^ mice exhibit a severe defect in thymic cortical architecture, including large epithelial free regions. Much of the epithelial component remains at an immature Keratin 5^+^ (K5) Keratin 8^+^(K8) stage, with a loss of defined cortical and medullary regions. A TOPFlash assay revealed a 2-fold increase in canonical Wnt signaling in TEC lines derived from *krm1*^− / −^  mice, when compared with *krm1*^+ / +^ derived TEC lines. Fluorescence activated cell sorting (FACS) analysis of dissociated thymus revealed a reduced frequency of both cortical (BP1^+^EpCAM^+^) and medullary (UEA-1^+^ EpCAM^hi^) epithelial subsets, within the *krm1*^− / −^  thymus. Surprisingly, no change in thymus size, total thymocyte number or the frequency of thymocyte subsets was detected in *krm1*^− / −^  mice. However, our data suggest that a loss of Krm1 leads to a severe defect in thymic architecture. Taken together, this study revealed a new role for Krm1 in proper development of thymic epithelium.


*Clinical & Developmental Immunology*, June–December 2006; 13(2–4): 299–319

Taylor & Francis

Taylor & Francis Group

### The Wnt signaling antagonist Kremen1 is required for development of thymic architecture

MASAKO OSADA[1], EMI ITO[1], HECTOR A. FERMIN[1], EDWIN VAZQUEZ-CINTRON[1], TADMIRI VENKATESH[1], ROLAND H. FRIEDEL[2], & MARK PEZZANO[1]

[1]*Department of Biology, The City College of the City University of New York, RCMI Center for the Study of the Cellular and Molecular Basis of Development, 138th Street and Convent Avenue, New York, NY 10031, USA, and* [2]*Department of Biological Sciences, Stanford University, 371 Serra Mall, Stanford, CA 94305, USA*

**Abstract**
Wnt signaling has been reported to regulate thymocyte proliferation and selection at several stages during T cell ontogeny, as well as the expression of *FoxN1* in thymic epithelial cells (TECs). Kremen1 (Krm1) is a negative regulator of the canonical Wnt signaling pathway, and functions together with the secreted Wnt inhibitor Dickkopf (Dkk) by competing for the lipoprotein receptor-related protein (LRP)-6 co-receptor for Wnts. Here *krm1* knockout mice were used to examine *krm1* expression in the thymus and its function in thymocyte and TEC development. *Krm1* expression was detected in both cortical and medullary TEC subsets, as well as in immature thymocyte subsets, beginning at the CD25+CD44+ (DN2) stage and continuing until the CD4+CD8+(DP) stage. Neonatal mice show elevated expression of *krm1* in all TEC subsets. *krm1* $^{-/-}$ mice exhibit a severe defect in thymic cortical architecture, including large epithelial free regions. Much of the epithelial component remains at an immature Keratin $5^{+}$ (K5) Keratin $8^{+}$(K8) stage, with a loss of defined cortical and medullary regions. A TOPFlash assay revealed a 2-fold increase in canonical Wnt signaling in TEC lines derived from *krm1* $^{-/-}$ mice, when compared with *krm1* $^{+/+}$ derived TEC lines. Fluorescence activated cell sorting (FACS) analysis of dissociated thymus revealed a reduced frequency of both cortical ($BP1^{+}EpCAM^{+}$) and medullary (UEA-$1^{+}$ $EpCAM^{hi}$) epithelial subsets, within the *krm1* $^{-/-}$ thymus. Surprisingly, no change in thymus size, total thymocyte number or the frequency of thymocyte subsets was detected in *krm1* $^{-/-}$ mice. However, our data suggest that a loss of Krm1 leads to a severe defect in thymic architecture. Taken together, this study revealed a new role for Krm1 in proper development of thymic epithelium.

**Keywords:** *Wnt signaling, Kremen1, T cell development, thymic organogenesis, thymic epithelium*

**Abbreviations:** *Dkk, Dickkopf; E, embryonic; FACS, fluorescence activated cell sorting; K5, keratin 5 expressing; K8, keratin 8 expressing;* krm1, *Kremen1; MHC, major histocompatibility complex; TCR, T cell receptor;* krm1$^{+/+}$, *wild type for both* kremen1 *alleles;* krm1$^{-/-}$, kremen1 *knockout;* krm1$^{+/-}$, kremen1 *heterozygous*

### Introduction

The thymus is a complex structure consisting of a mesenchyme-derived capsule surrounding a network of stromal components, which include endoderm-derived epithelial cells, hematopoietic-derived thymocytes, macrophages, dendritic cells and blood vessels. The thymus is central to a properly functioning immune response and its functions include recruitment of T cell progenitors from the bone marrow, commitment of precursors to the T cell lineage, expansion of immature subsets, MHC restriction and T cell repertoire selection. These unique functions are primarily the result of interactions between developing thymocytes and the microenvironments defined by the thymic epithelial components of the stroma. The

Correspondence: M. Pezzano, Department of Biology, The City College of the City University of New York, RCMI Center for the Study of the Cellular and Molecular Basis of Development, 138th Street and Convent Avenue, New York, NY 10031, USA. Tel: 1 212 650 8559. Fax: 1 212 650 7989. E-mail: mpezzano@sci.ccny.cuny.edu

ISSN 1740-2522 print/ISSN 1740-2530 online © 2006 Taylor & Francis
DOI: 10.1080/17402520600935097

thymic stroma is roughly divided into two regions on the basis of histology, the cortex and the medulla. The TECs in each region are functionally and phenotypically distinct, with cortical types being defined by expression of keratin 8 (K8) and MHC II, together with the surface markers CDR1, BP1 and DEC205. In contrast, medullary types are defined by expression of keratin 5 (K5) and MHC II together with MTS10, *Ulex europaeus* agglutinin lectin binding (UEA-1$^{+}$) and CD80. A minor subset of $K8^{+}K18^{+}K5^{+}K14^{-}$ has been reported in the cortex, and resides at the corticomedullary junction (Klug et al. 1998; 2000). All of the epithelial cells appear to be derived from a common progenitor population (TEPC) that expresses MTS24, together with both K5 and K8 (Gill et al. 2002). This population dominates the epithelial component at E10.5–12.5, but later becomes restricted to a small population that is maintained in the medulla (Bennett et al. 2002). K8 expression becomes restricted to cortical cells, while K5 is restricted to medullary cells by E13; however, reduced numbers of K5+K8+ cells persist primarily at the corticomedullary junction. While the lineage relationships between the different populations and the molecular mechanisms that control their differentiation are not well defined, the cortical and medullary epithelial cells appear to have distinct roles in the attraction, expansion and differentiation of developing thymocytes.

The thymic cortex represents a unique thymic microenvironment where developing immature thymocytes are held in a complex 3D reticular network of MHCII$^{+}$ epithelial cells. The cortical epithelium is responsible for the attraction of T cell precursors, commitment to the T cell lineage and expansion of immature DN thymocytes, ultimately resulting in the generation of a large pool of DP thymocytes expressing a diverse unselected $\alpha/\beta$ T cell receptor (TCR) repertoire. Recognition of self-peptide/MHC complexes by a subset of these thymocytes then rescues that population from apoptosis (positive selection) (Savage and Davis 2001). The proper formation of this key thymic microenvironment is dependent on interactions between developing thymocytes and immature thymic epithelial cells (TECs) called thymic crosstalk (van Ewijk et al. 1999; 2000). A key step in understanding cortical epithelial development will be to define the cell surface molecules and/or soluble factors produced by thymocytes and epithelium, which contribute to "crosstalk" and effect the development and organization of the thymic cortex. Equally important will be understanding the signaling pathways which regulate the epithelial/mesenchyme communication important for thymic organogenesis and TEC development.

The thymic medulla is composed of a heterogeneous population of epithelial cells that provide a microenvironment for newly positively selected CD4 and CD8 SP thymocytes. Like the cortex, proper organization of mTECs may require crosstalk from SP thymocytes (Shores et al. 1991; Surh et al. 1992) as well as $\gamma/\delta$ T cells (Ferrick et al. 1989). MTECs, acting together with MHC class II$^{+}$ dendritic cells residing at the corticomedullary junction, function to negatively select thymocytes which bear self-reactive TCRs (Barclay and Mayrhofer 1981). MTECs also express a wide array of tissue specific genes, (TSAs) so-called "promiscuous gene expression" (Gotter et al. 2004; Kyewski and Derbinski 2004), some of which appear to be under the control of the AIRE transcription factor (Anderson et al. 2002), and may represent a pool of self-antigens used to negatively select autoreactive thymocytes or induce differentiation of regulatory subsets. In addition to their role in tolerance induction, mTECs may also regulate post selection differentiation events including up-regulation of CD69, CD24 and CD62L as well as expansion of SP thymocytes prior to their export from the thymus (Gabor et al. 1997).

The signaling mechanisms that control early thymic organogenesis are beginning to be unraveled (for reviews see Manley (2000)). The later events of this process, involving TEC differentiation and expansion, are under the control of FoxN1. The loss of FoxN1 (Whn, the defect found in nude mice) causes an early arrest in TECs development and expansion, and a loss of the capacity to attract hematopoietic precursor cells (Blackburn et al. 1996). Transcription control of FoxN1 expression was recently shown to be regulated by secreted Wnt proteins, expressed by TECs and thymocytes (Balciunaite et al. 2002). This lead us to test the hypothesis that regulation of Wnt signaling would prove to be critical to proper thymic stromal organization or the development of TEC populations.

The current model of canonical Wnt signaling involves soluble Wnts binding to a co-receptor composed of seven transmembrane spanning Frizzled (Fz) proteins, together with low-density LRPs-5 and -6 (Tamai et al. 2000). Wnt receptor binding is highly regulated through association with diverse secreted proteins including Dickkopf (Dkk) (Niehrs 1999), Frzb-1 (Leyns et al. 1997)or Cerberus (Leyns et al. 1997), as well as extracellular and cell-membrane glycosaminoglycans (Wodarz and Nusse 1998). Wnt signals are transduced through at least three different intracellular signaling pathways, with the canonical Wnt pathway being the most studied and appearing to be the most critical to T cell development. The canonical Wnt pathway leads to stabilization of $\beta$-catenin and its subsequent translocation to the nucleus, where it engages with the lymphoid enhancer factor (LEF)-1 as well as the T-cell factors (TCF)-1, -3 or -4. The binding of $\beta$-catenin to LEF or TCF turns these proteins into active transcription factors for genes, which modulate cell fate, proliferation and survival during embryonic development, including the

T cell-specific genes *CD3ε*, *TCRα*, *TCRβ*, *CD4* and *TCRγ* (for review see Miller (2002)). A key role for the Wnt signaling cascade in controlling thymocyte cellularity and differentiation is apparent from Tcf-1/LEF-1 knockout studies, as well as a number of complementary gain-of-function and loss-of-function experiments performed with upstream components of the canonical pathway (Hattori et al. 1996a,b; Schilham et al. 1998; Ioannidis et al. 2001; Staal et al. 2001). Differential expression of *Wnt* genes was also observed in distinct maturation stages of TECs, implying a possible role for Wnt signaling in the proper development of the thymic stroma, as well (Balciunaite et al. 2002). Epithelial cells express even higher levels of Wnts, including Wnt-1 and this may contribute to the thymic crosstalk necessary for both thymocyte and stromal development. Separation of thymocytes from Wnt-producing epithelial cells and the thymic micro-environment triggers β-catenin phosphorylation and degradation in the thymocytes (Pongracz et al. 2003). All experimental models that impair Wnt signaling, result in reductions in thymocyte numbers or blocks in development, however, defects in thymic architecture have not been previously reported.

Dkk proteins are soluble molecules that bind to and inactivate the LRP6 co-receptor for Wnts (Mao et al. 2001). Dkk proteins act through transmembrane proteins, Kremen1 (Krm1) and Kremen2 (Krm2), which are high affinity receptors for Dkk1 (Davidson et al. 2002; Li et al. 2002; Mao et al. 2002). The membrane-anchored molecule Krm binds the Dkk/LRP6 complex, triggering internalization and clearance from the cell surface (Mao et al. 2002). Given that LRP6 only mediates the canonical pathway of Wnt signaling, Dkk and Krm1 can be considered specific inhibitors of the canonical Wnt signaling pathway. Loss of Krm1 should result in excessive canonical Wnt signaling. In studies performed to examine the role of Kremen proteins and Dkk during *Xenopus* embryogenesis, Krm proteins were shown to function in a Wnt inhibition pathway regulating early anterior–posterior patterning of the CNS. Knock-down of Krm1 and Krm2 with antisense morpholinos leads to a deficiency of anterior neural development, while treatment with Wnt8 posteriorises embryos (Davidson et al. 2002).

In this study, we examined thymic epithelial organization and development in a mouse in which the *krm1* gene had been disrupted through the use of targeted gene trapping (Friedel et al. 2005). We show that loss of *krm1* results in increased canonical Wnt signaling. We examined the expression pattern of *krm1* in both thymocyte subsets and the epithelial components of the thymus. The effects of loss of *krm1* on thymocyte development, as well as the development and organization of thymic architecture are reported. Our results suggest that regulation of Wnt signaling by Krm1 is critical during thymic organogenesis, to allow development of a properly organized epithelial component of the thymic stroma.

### Methods

### *Mice*

*Krm1* $^{-/-}$ mice were generated by targeted gene-trapping as described previously (Friedel et al. 2005) and then backcrossed to C57BL/6 mice (Jackson Labs). Mice were bred and used for this study in accordance with protocols approved by the City College IACUC. The age of the mice used for these experiments was between 5 day neonatal and 8 weeks of age, as described for specific experiments.

### *Genotyping of Kremen1 KO mice*

For genotyping, DNA was extracted by treating either tail snips or ear punch fragments with 100 μl of 50 mM NaOH for 20 min at 100°C. To each sample, 30 μl of 1 M Tris pH 7.5 was added and cooled on ice. Undigested material was removed by centrifugation at high speed in a microfuge. About 5 μl of the resulting samples was subjected to PCR using the following set of primers, Kremen 1 forward: 5′ GCA GACACA TGT TGG GAT ATT GGC 3′, Kremen 1 reverse: 5′ TGA GGG AGC AGT GTG AAG AGT TCT 3′, trapping vector: 5′ CT TCC GGA GCG GAT CTC AAA CTC 3′. Amplification of WT samples results in two copies of a 750 bp product, while KO samples result in two copies of a 550 bp fragment. Heterozygous mice yield two fragments of 550 and 750 bp.

### *Thymic epithelial cell lines*

The primary TEC lines used in this study were created by dissociating thymic tissue, as described above (Gray et al. 2002) and allowing the resulting cells to adhere to tissue culture dishes in RPMI with 10% FBS. The resulting mixed cultures were expanded for ten passages, before cloning by limiting dilution. The resulting clones were then expanded, and 4 WT and 4 KO derived epithelial lines were selected for further study, based similar morphology and growth characteristics, together with expression of keratin, using a pan anti-mouse keratin antibody (Sigma).

### *RT-PCR*

RNA from primary TECs, derived from Krm1 WT and KO mice, were isolated using RNAquous-4PCR kit (Ambion). First strand cDNA was generated from 1 μg of RNA using Retroscript (Ambion) and PCR analysis was performed using gene specific primers for Krm1, FW 5′-CAG CGC TGC AAG GTG GGA AGC-3′ and RV 5′-GAT ATC TCC AGA AGC CCC AGG CTG ACG-3′. Gene specific primers for

β-actin, FW 5′-GTT ACC AAC TGG GAC GAC A and RV 5′-TGG CCA TCT CCT GCT CGA A-3′ were used for internal control. Gene specific primer sequences for Wnt1, Wnt4, Fz1, Fz2, Fz3, Fz4, Fz5, Fz6, Fz7, Fz8, Fz9, Fz10, LRP6, Tcf-1, Lef-1 and β-catenin (Pongracz et al. 2003) as well as Dkk2 and Dkk3 (Mao and Niehrs 2003), were described previously. Products were run on 2% agarose gels and visualized by ethidium bromide.

*TOPFlash assay*

Primary TEC lines, derived from Krm1 WT and KO mice, were transfected in six well plates with 20 ng of pRL-TK (Promega) and 1 μg of TOPFlash plasmid (Upstate Biotechnology Inc.) using Lipofectamine2000 transfection reagent (Invitrogen). Forty-eight hours after transfection, cells were rinsed once with PBS and cell lysates were prepared using passive lysis buffer (Promega). Luciferase assays were performed using the Dual Luciferase assay kit (Promega). Firefly luciferase activity was normalized to *Renilla* luciferase activity for each sample and expressed as relative luciferase activity. This allows normalization for transfection efficiency. Experiments were performed in triplicate and repeated more than four times with the four different TEC clones from each mouse strain.

In situ *hybridization*

Frozen thymus sections (10 μm) from a wild type neonatal mouse were fixed in 4% PFA for 10 min at room temperature (RT) and washed in PBS (Ca and Mg free) for 3 min three times. The sections were acetylated for 10 min at RT in freshly made acetylation solution (300 μl of 0.1 M triethanol amine pH 7 with 800 μl of acetic anhydride), washed in PBS (Ca and Mg free) for 3 min three times, and incubated for 2 h at RT in hybidization buffer (50% formamide, 5× SSC, 5× Denhardt's, 250 μg/ml MRE 600 tRNA, 500 μg/ml herring sperm DNA). Full length *Krm1* antisense and sense probes were generated from a *Krm1* cDNA containing plasmid (generous gifts from Dr Christof Niehrs, Division of Molecular Embryology, Deutsches Krebsforschungszentrum and Dr Nakamura, Osaka University) by *in vitro* transcription (MAXI script® SP6/T7 *in vitro* transcription kit, Ambion Inc.). The probes were labeled with digoxigenin using the DIG RNA labeling mix (Roche). The probes were diluted to 300 μg/ml in hybridization buffer and were denatured at 80°C for 8 min and then immediately cooled on ice. The sections were incubated overnight at 65°C in a moisture chamber. The slides were washed in 5× SSC for 10 min at 65°C. The sections were washed five times in 0.2× SSC for 30 min at 65°C, and then once in 0.2× SSC for 5 min at RT. After incubation in 0.15 M NaCl/0.1 M Tris pH 7.5 buffer for 5 min at RT, the sections were prehybridized in 10% heat-inactivated sheep serum, diluted in 0.15 M NaCl/0.1 M Tris pH 7.5 buffer, for 1 h at RT. Slides were then incubated overnight a 4°C with anti-DIG-AP, Fab fragment (Roche) diluted 1:3500 in 10% heat-inactivated sheep serum/0.15 M NaCl/0.1 M Tris pH 7.5 buffer. The sections were washed in 1% sheep serum/0.15 M NaCl/0.1 M Tris pH 7.5 buffer three times for 15 min each followed by one wash for 15 min at RT in 0.1% Triton X100/0.15 M NaCl/0.1 M Tris pH 7.5 buffer. Sections were equilibrated in 50 mM $MgCl_2$/0.1% Triton X100/0.1 M NaCl/0.1 M Tris pH 9.5 buffer for 5 min at RT and incubated with NBT/BCIP solution (Roche) diluted 1:50 in the same buffer for 6 h at RT in the dark. The slides were washed in TE-buffer, then in water, and fixed in 4% PFA for 30 min at RT. The slides were mounted using Aquapolymount (Polysciences Inc.) and observed using a Nikon TE2000 inverted microscope.

*X-gal staining*

Thymi from freshly sacrificed *krm1*$^{+/-}$ mice were removed and frozen cryostat sections (8 μm) were prepared. Sections were fixed with 3% paraformaldehyde for 10 min and washed three times with PBS for 10 min each time. Sections were stained with X-gal (1 mg/ml) in staining buffer (0.1 M phosphate buffer pH7.3, 2 mM $MgCl_2$, 5 mM $K_4Fe(CN)_6$, 5 mM $K_3Fe(CN)_6$, 0.02% NP-40, 0.1% sodium deoxycholate) for 12 h at 37°C followed by washing three times with 1× PBS and post fixed with 3% paraformaldehyde. Slides were mounted with VectaMount (Vector Laboratories) and observed using a Nikon TE2000 inverted microscope.

*Tissue dissociation*

Thymus tissue was dissociated as described in Gray et al. (2002). Briefly, thymi were removed from freshly sacrificed mice and cut into small pieces with scissors. Thymocytes were released in 5 ml RPMI by gentle agitation using a magnetic stir bar for 10 min on ice. The process was repeated two to three times until the majority of thymocytes were released. The remaining tissue fragments were subjected to enzymatic digestion in collagenase D (1.25 mg/ml) and DNAse I (0.1 mg/ml) (Roche applied science) in RPMI at 37°C for 15 min with gentle agitation every 5 min. Dissociated cells were transferred to the new tube and fresh enzymatic digestion medium were added. This procedure was repeated three times. Remaining small fragments were passed through a 26G needle and then through a 100 μm cell strainer (BD biosciences). The cell suspension was washed once with PBS and resuspended in FACS staining buffer (FSB) containing PBS + 3% FCS + 0.02% $NaCN_3$.

*FACS-gal and flow cytometry analysis*

Freshly isolated thymocytes and stromal cells from *krm1* $^{+/+}$ and *krm1* $^{-/-}$ mice were washed once with PBS. About $2 \times 10^6$ cells were resuspended in 50 µl of pre-warmed RPMI with 10% FBS at 37°C. Fluorescein-di-β-galactopyranoside (FDG) was diluted to 2 mM in water and pre-warmed at 37°C. About 50 µl of FDG solution were added to the cells and incubated at 37°C for exactly 1 min for osmotic loading of FDG. FDG loading was stopped by addition of 900 µl of ice-cold RPMI with 10% FBS. FDG loaded cells were then incubated with various primary antibodies specific to cell surface markers for 20 min on ice. Cells were washed once with FSB and incubated with secondary antibody for 20 min on ice. Labeled cells were washed once with FSB and resuspended in 400 µl FSB prior to analysis using a BD LSRII flow cytometer. Two *krm1* $^{+/+}$ and two *krm1* $^{-/-}$ thymi were pooled for each analysis to yield an adequate number of epithelial cells for multiple FACS analyses. Viable cells were gated based on their forward and side scatter profile. For the TEC analysis, only $CD45^-$ populations, which are free of hematopoietic cells, were analyzed for expression of BP1 or CDR1 together with either MHCII or EpCAM to identify cortical epithelial subsets. Alternatively, UEA-1 or CD80 was used in combination with EpCAM or MHCII to identify medullary subsets. The frequency of $FDG^+$ cells in each of the gated TEC subsets represents the frequency that express *krm1*. TEC enriched fractions from dissociated *krm1* $^{+/+}$ thymi were stained and analyzed using the same FACS-gal assay to provide a negative control to allow detection of true β-gal reporter gene expression in *krm1* $^{-/-}$ mice over background from endogenous β-gal activity.

*Antibodies and immunoconjugates*

Primary antibodies used for flow-cytometry analysis and/or immunohistochemistry were APC or PE Cy7-conjugated anti-CD4 (clone RM4-5, BD Pharmingen), FITC or PE-conjugated anti-CD8 (clone H35-17.2, BD Pharmingen), PerCP Cy5.5 conjugated anti-CD25 (clone PC61, BD Pharmingen), biotin conjugated anti-CD44 (clone IM7, BD Pharmingen), APC or biotin conjugated anti-Thy1.2 (BD Pharmingen), APC conjugated anti-TCRβ (clone H57-597, BD Pharmingen), APC conjugated anti-CD45 (clone 30-F11, BD Pharmingen), PE conjugated anti-I-A/I-E-PE (M5/114.15.2, BD Pharmingen), PE-conjugated anti-CD80 (clone 16-10A1, BD Pharmingen), PE-conjugated anti-BP1 (clone FG35.4, eBioscience), anti-mouse K8 (TROMA1, Developmental Hybridoma Bank), polyclonal Rabbit anti-pan Keratin (DAKO), PE-conjugated UEA1-lectin (Biomeda), and anti-mouse K5 (MK5, Covance Research Products). The CDR1 and G8.8 antibodies were purified from hybridoma supernatant obtained from the Developmental Hybridoma Bank and Biotinylated using EZ-link NHS-Biotin (Pierce) according to the manufacturer's instructions. NDLC145 (anti-DEC205) antibody was purified from hybridoma supernatant (Generous Gift from Dr R. Steinman, Rockefeller University). The secondary reagents used were FITC-conjugated anti-rat IgG2a and APC Cy7-conjugated streptavidin (BD Pharmingen), TRITC-conjugated goat anti-rabbit IgG (Jackson Immunoresearch), and anti-rat IgG-FITC (Sigma).

*Immunohistochemistry*

Thymi from freshly sacrificed *krm1* $^{+/+}$, *krm1* $^{+/-}$ and *krm1* $^{-/-}$ mice were removed and frozen cryostat sections (8 µm) were prepared. Sections were air-dried for 10 min and fixed with ice-cold acetone for 2 min, followed by three washes with PBS for 5 min. Sections were incubated with 100 µl of a mixture of optimally diluted primary antibodies diluted in 1× PBS with 3% FBS for 15 min at RT. Sections were washed three times with PBS for 5 min prior to incubation with 100 µl of secondary antibody diluted in PBS with 3% FBS for 15 min at RT. Slides were again subjected to three washes with PBS for 5 min. Sections were mounted in ProLong Gold anti-fade reagent with DAPI (Molecular Probes) and observed using a Zeiss 510 confocal microscope or a Nikon TE2000 inverted microscope equipped with epi-fluorescence and a SPOT digital camera system.

### Results

*The Kremen-1 KO mouse*

The Krm1 knockout mouse was created using a targeted gene trapping strategy. This strategy employed the PLAP secretory trap vector, which was flanked by homology arms to facilitate insertion into the fourth intron of the *Krm1* gene, effectively trapping the down stream exons, while placing the β-gal and PLAP reporter genes under the control of the Krm1 promoter. A bicistronic transcript encodes two proteins, a fusion of the endogenous protein and β-geo, retained in cell bodies; and PLAP protein, localized on axonal membranes (Friedel et al. 2005). Proper insertion of the vector into the ES cells, used to create the knockout mouse line, was confirmed by Southern blot (Friedel et al. 2005), while subsequent maintenance of the insertion in the mouse lines was confirmed using PCR-based genotyping (Figure 1A). The initial mouse lines, created by injection of the targeted ES cells into CD1 blastocysts, were backcrossed for seven generations to

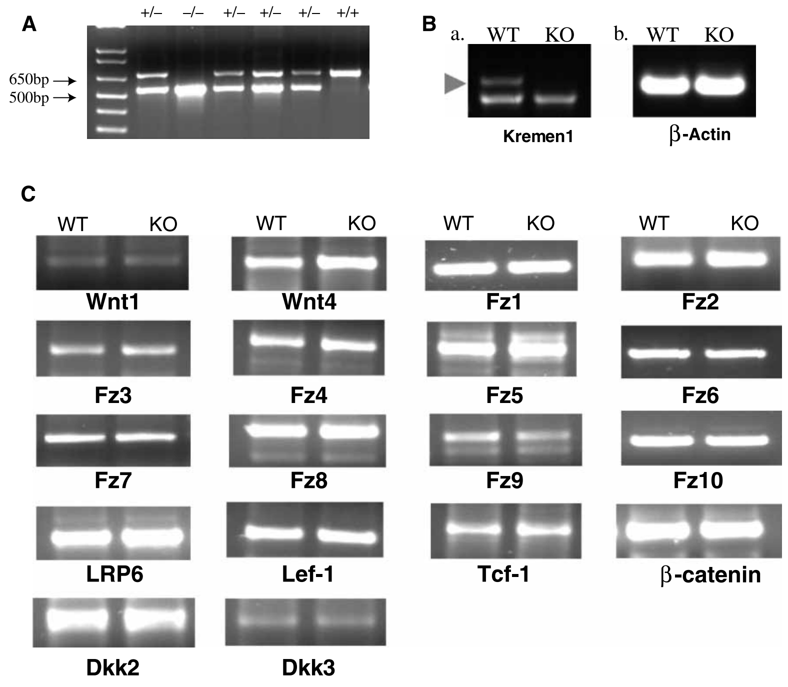

Figure 1. (A) PCR genotyping of *Krm1* KO mice. DNA extracted from tail snips was subjected to PCR using primers specific to the Krm1 intron sequence as well as primers specific to the trapping vector. Amplification of WT samples results in two copies of the upper 750 bp product, while KO samples result in two copies of the lower 550 bp fragment. Heterozygous mice yield two fragments of 550 and 750 bp. (B) *krm1* expression is lost in the *krm1* KO mouse. To confirm loss of Krm1 gene expression in the KO mouse line, total RNA from TEC lines derived from *Krm1* WT and KO mice were subjected to RT-PCR analysis to detect full length *Krm1* mRNA. (a) Detection of Krm1. Sequence analysis of the PCR products revealed that the upper band (gray arrow) is *Krm1*, whereas the lower band is a non-specific PCR product. (b) Detection of β-actin from the same samples as an internal control. (C) Expression of components of the Wnt signaling pathway in TEC lines derived from *krm1WT* and *krm1KO mice*. RT-PCR was used to confirm the expression of Wnt 1, Wnt 4, the Wnt co-receptor LRP6, together with the Wnt receptors Fz1–10, the transcription factors Lef-1 and Tcf-1, β-catenin and the soluble inhibitors Dkk2 and Dkk3.

C57BL/6 by breeding krm1$^{+/-}$ mice to C57BL/6 WT animals. The resulting line is maintained by back-crossing krm1$^{+/-}$ animals to C57BL/6 WT mice. All experiments were performed, wherever possible, using liter mates of krm1$^{+/-}$ × krm1$^{+/-}$ matings, to minimize the influence of genetic background on the observed phenotypes. The resulting *Krm1* KO mice appear normal on external and gross internal evaluation. They have normal sized organs, with no gross abnormalities or size differences apparent by H and E staining of histological sections (data not shown), and produce the expected frequency of offspring in crosses, suggesting that the loss of *Krm1* and the resulting increase in Wnt signaling, results in no gross developmental defects, that might indirectly influence thymic organogenesis. To confirm the loss of *Krm1* gene expression in the KO mouse line, total RNA from TEC lines derived from *Krm1* WT and KO mice, were subjected to RT-PCR analysis, to detect full length *Krm1* mRNA. A full-length *Krm1* PCR product was only detected in the WT derived TEC cell line (Figure 1B, panel a, arrow), while no band was detected in the KO-derived TEC cell line. Sequence analysis of the PCR products revealed that upper band (arrow) is *Krm1*, whereas the lower band is a non-specific PCR product. Identical results were obtained with RNA derived from whole thymus and purified thymocytes (data not shown).

### *The canonical Wnt signaling components are present in the TECs of* krm1 $^{-/-}$ *mice*

The presence of the signaling components, necessary to allow canonical Wnt signaling in the *krm1* $^{-/-}$ mice, was confirmed using RT-PCR to amplify mRNA derived from both WT and KO primary TEC lines. Expression of the co-receptors for Wnts, LRP6, Fz1, Fz2, Fz3, Fz4, Fz5, Fz6, Fz7, Fz8, Fz9 and Fz10 was detected in, both *krm1* $^{+/+}$ and *krm1* $^{-/-}$ mice, together with low levels of *Wnt1* and strong expression of *Wnt4*. (Figure 1C). For Krm1 to function as an inhibitor of the canonical Wnt signaling pathway, it must interact with soluble Wnt inhibitors in the Dkk family. Using RT-PCR, we demonstrated that both Dkk2 and Dkk3, but not Dkk1 are expressed in both WT and KO TEC lines. We also confirmed expression of the down stream components of the canonical pathway, β-catenin, Lef-1 and Tcf-1, in both the WT and KO TEC lines (Figure 1C). Taken together these data demonstrate that the TECs, within both WT and Krm1 KO mice, are capable of canonical Wnt signaling, possibly mediated by Wnts secreted by the

TECs themselves. No effect of loss of *krm1* was apparent, for expression of key transcription factors known to influence TEC development, including AIRE, Foxn1, Hoxa3 or Pax1 analyzed by RT-PCR of TEC enriched samples from dissociated thymus (data not shown).

### *Loss of Kremen1 results in increased canonical Wnt signaling in thymic epithelial cells*

To directly measure the effect of the loss of *Krm1*, on endogenous canonical Wnt signaling, primary TEC lines were transfected with a TOPFlash reporter construct, prior to performing a TOPFlash assay. The TOPFlash assay measures β-catenin/TCF driven expression of a luciferase reporter gene. A 2-fold increase in endogenous Wnt signaling was detected within TECs derived from *krm1* $^{-/-}$ mice, when compared to *krm1* $^{+/+}$ derived TECs (Figure 2) ($p =$ 0.001). This increase in canonical Wnt signaling is representative of five experiments, and reflects a similar increase observed in four independent TEC lines, derived from each mouse strain. This demonstrates that the observed increase in Wnt signaling in not merely a TEC clone specific effect.

### *Kremen1 expression in the thymus*

*In situ* hybridization of thymic sections, derived from 5-day neonatal *krm1* $^{+/+}$ mice, with a full-length *krm1* anti-sense RNA probe, revealed strong *krm1* expression in the thymic cortex (Figure 3A and inset) with limited expression in discrete cells or small clusters in the medulla. No hybridization was observed in sections hybridized with a full-length *krm1* sense probe (Figure 3B). Similar hybridizations, with sections derived from adult thymus, showed a reduction in cortical hybridization, while maintaining strong but discrete expression in the medulla (data not shown), similar to the pattern observed when X-gal staining was used (Figure 3F). A β-gal reporter gene, under the control of the krm1 promoter, was used to monitor tissue expression of *krm1* in whole mounts, as well as cryostat sections of fresh thymus from *krm1* $^{-/-}$, *krm1* $^{+/-}$ and *krm1* $^{+/+}$ animals. The specificity of the β-gal reporter, over endogenous β-gal activity, could be seen in the X-gal stained (indicated by blue color) *krm1* $^{+/+}$, *krm1* $^{+/-}$ and *krm1* $^{-/-}$ thymic whole mounts, shown in Figure 3C. A comparison of the size and morphology of the lobes, indicates that there were no major defects in the development of the thymus in *krm1* deficient mice. Strong blue X-gal staining was apparent in the *krm1* $^{-/-}$ thymic lobe (panel C middle), whereas reduced staining could be seen in the *krm1* $^{+/-}$ thymic lobe (panel C left, where only one copy of the β-galactosidase reporter gene is present), while no detectable staining was observed in the *krm1* $^{+/+}$ lobe (panel C right), indicating that *krm1* is broadly expressed in the thymus of 1-month-old mice. When thymic sections from *krm1* $^{+/-}$ mice were analyzed, intense X-gal staining was visible in a variety of cell types (Figure 3F) with strong staining restricted in its distribution. The arrows in panel F show intensely stained cells localized to the medulla in adult *krm1* $^{+/-}$ mice. In contrast, X-gal staining of 5-day old *krm1* $^{+/-}$ neonatal mice, revealed a more extensive distribution of *krm1* expressing cells in the subcapsular cortex and

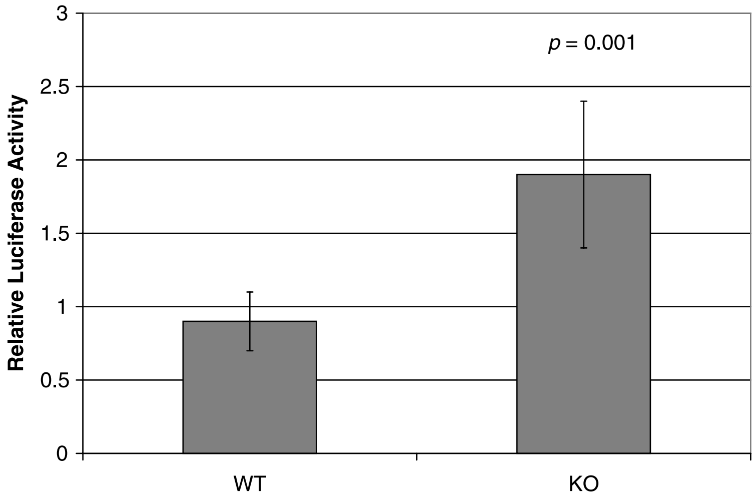

Figure 2. Increased endogenous canonical Wnt signaling activity in KO-TEC lines, measured by the TOPFlash assay. TEC lines derived from Krm1 WT and KO mice were transfected with the TOPFlash and pRL-TK plasmids to measure endogenous Wnt signaling activity. Renilla Luciferase activity was used as a transfection control. TOPFlash activity was normalized for Renilla luciferase activity in each sample and shown as a relative luciferase activity. Means shown represent an average relative luciferase activity, calculated for four different TEC clones derived from each mouse strain. Bars represent a mean of five experiments calculated with triplicate wells +/− SD. *-The $p$-value (0.001) was determined using a Student $t$-test to compare the mean relative luciferase activities calculated for wild type and knockout epithelial cell lines.

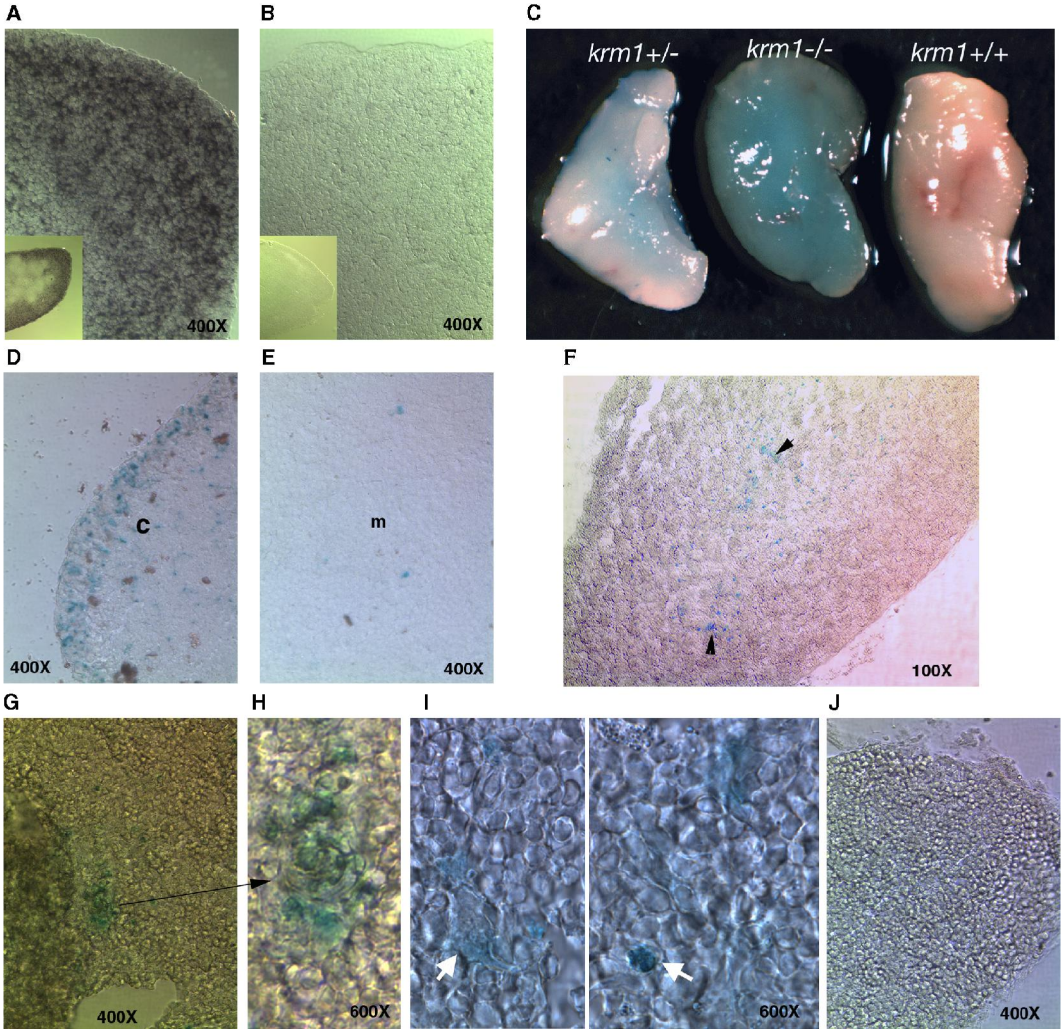

Figure 3. Expression of *krm1* in the thymus. *Krm1* expression in the thymus of 5-day old neonatal mice demonstrated by *in situ* hybridization with a DIG labeled *krm1* anti-sense RNA probe (A). The inset shows a 100x magnification of the same section. Note the expression is primarily localized to the cortex; (B) *krm1* sense probe. *Krm1* expression was detected in *krm1* $^{-/-}$ mice using X-gal to stain cells, which express a β-gal reporter gene under the control of the *krm1* promoter. Whole mount staining of the thymus from *krm1* $^{-/-}$ mice (C middle lobe) showed uniform strong blue X-gal staining indicating that k*rm1* is broadly expressed in 1 month old mice. The Krm1$^{+/-}$ thymus (C, left lobe) showed weaker staining since it carries only one copy of the reporter gene. No X-gal staining was visible in the *krm1* $^{+/+}$ littermate thymus (C, right lobe) indicating that there was no endogenous β gal activity detected in the assay. No difference in thymus size or morphology was apparent. Cryostat sections of adult thymus reveal strong *krm1* expression in the medulla of adult mice (F, black arrows). In contrast neonatal mice exhibit strong X-gal staining in the cortex (C) with only limited staining in the medulla (D). Examination of sections from adult mice at higher magnification allowed localization of X-gal staining within cells with an epithelial morphology (G–I, white arrow), as well as cells with a lymphoid morphology (J, white arrow). (K) *krm1* $^{+/+}$ control.

cortex (Figure 3D) with more limited expression in the medulla (Figure 3E). This would suggest higher *krm1* expression during earlier stages of thymus development, when both thymocytes and epithelial cells are expanding in numbers. In both adult and neonatal mice, expression of *krm1* appeared in cells with an epithelial morphology (Figure 3G–I, white arrow) and lymphoid morphology (Figure 3J, white arrow). No endogenous β-gal activity was detectible in *krm1* $^{+/+}$ sections stained under the same conditions (Figure 3K).

It is difficult to identify specific cell types in thymic sections expressing krm1, simply by morphology, using *in situ* hybridization or X-gal staining. The fluorescent β-gal substrate (FDG) was used, together with cell surface antibody staining and FACS analysis, to specifically identify the subsets of epithelial cells (Figure 4) and thymocytes (Figure 9A–B) in the thymus, which express *krm1* (based on the *krm1* promoter driven β-gal activity). The profile of TECs from *krm1* $^{+/+}$ to *krm1* $^{-/-}$ used for gating the various TEC subsets is provided on the left of each panel in Figure 4, after gating on CD45$^{-}$ cells to enrich for the TEC subsets. A comparison of FDG staining in TECs of 5-day neonatal mice, compared with 8-week old adult animals, reveals a greater frequency of both cTECs and mTECs expressing *krm1* in neonatal animals (Figure 4A and B-blue lines in histograms)

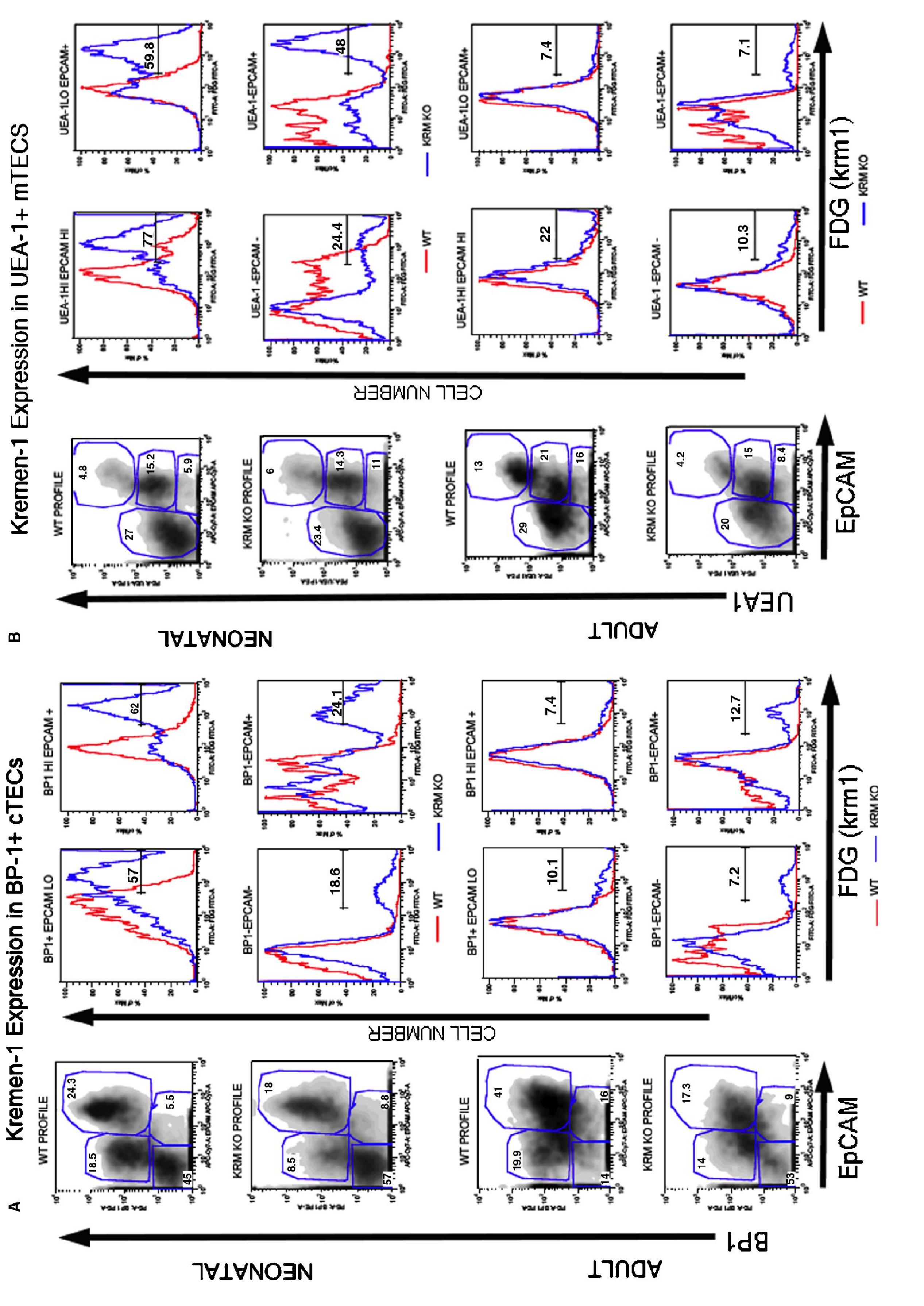

A

Kremen-1 Expression in BP-1+ cTECs

NEONATAL

ADULT

BP1

EpCAM

CELL NUMBER

FDG (krm1)

WT PROFILE

KRM KO PROFILE

BP1+ EPCAM LO

BP1 HI EPCAM +

BP1-EPCAM-

BP1-EPCAM+

WT

KRM KO

B

Kremen-1 Expression in UEA-1+ mTECS

UEA1

UEA-1HI EPCAM HI

UEA-1LO EPCAM+

UEA-1 -EPCAM -

UEA-1-EPCAM+

with the highest frequency of *krm1* expression found in the mature medullary subsets defined by high expression of UEA-1 together with EpCAM (77%, Figure 4B). A very high frequency of *krm1* expressing cells was also detected in neonatal mice among the cortical TEC subsets, defined by BP-1 together with EpCAM (62%, Figure 4A). When 8-week-old adult mice were assayed, the frequency of *krm1* expressing medullary subsets dropped to 22% for the UEA-$1^{hi}$ $EpCAM^{hi}$ (Figure 4A) TEC subset. Most of the UEA-$1^{hi}$ $EpCAM^{hi}$ cells were so brightly stained, that they saturated the histogram to the right. These extremely bright cells were never present in *krm1* $^{+/+}$ controls, and may represent the small population of cells that appear as very strongly stained cells, identified by arrows in Figure 3F, using the less sensitive X-gal assay. For the cTEC subsets defined by BP1 (Figure 4B) the frequency of *Krm1* expressing cells drops to less than 10%. This was consistent with the X-gal stained sections, which showed almost no β-$gal^{+}$ cells in the cortex of adult mice (Figure 3F), while strong staining was detected in sections prepared from 5-day neonatal animals (Figure 3D). The results presented in Figure 4 are representative of the results obtained in three identical experiments. These results clearly demonstrate that *krm1* is expressed in a large percentage of both cortical and medullary TEC populations in the neonatal mouse, with a smaller population of TECs continuing to express *krm1* in adult animals. Given the increase in canonical Wnt signaling observed in the *krm1* $^{-/-}$ TECs (Figure 2), together with strong krm1 expression and altered profiles in both mTEC and cTECs detected by FACS, we examined the effect that loss of *krm1* would have on the development of the thymic architecture and the distribution of distinct TEC subsets within the thymus. We chose to present the data obtained from *krm1* $^{-/-}$ animals in Figure 4 rather than hemizygous animals, to allow a comparison of the frequency of the different cortical and medullary subsets in *krm1* $^{-/-}$ and *krm1* $^{+/+}$ animals, as well as the gating used for determining *krm1* expression in those subsets. The profile of TEC profile for hemizygous animals was very similar to that observed in *krm1* $^{-/-}$ mice. While all of the TEC subsets are present in *krm1* $^{-/-}$ animals, there is a dramatic reduction in the frequency of the BP1+EpCAM+cTEC and UEA-1+EpCAM+ mTEC populations, when compared to WT mice (see profiles on the left of each panel in Figure 4). This is most apparent in the adult profile comparisons, where the frequency of BP1+EpCAM+cTECs was 17% in the KO, compared to 41% in the WT animals; the frequency of the UEA-1+EpCAM+mTECs was 4% in the KO, compared to 12% in WT mice. A smaller reduction in cTECs (16% KO vs. 24% WT) was apparent in neonatal mice; however, the frequency of mTECs was unchanged. We also observed a small but consistent decrease in the frequency of the $BP1^{+}$ $EpCAM^{-}$ population, in both neonatal and adult *krm1* $^{-/-}$ mice. The majority of this population (with the exception of a small fraction of CD31+ endothelial cells) represents the recently described cortical mesenchyme population (Muller et al. 2005). The differences in the TEC profiles shown for Figure 4 are representative of the results obtained for five independent experiments. The inherent variation in the method used to dissociate the lobes and the fact that multiple lobes were required to acquire enough cells for analysis, makes statistical analysis using this technique difficult. Similar reductions in TEC frequencies were obtained using *krm1* $^{+/-}$ animals (data not shown), suggesting that loss of a single *krm1* allele is sufficient to affect the frequency of TEC subsets. These differences in cTEC and mTEC frequency were also strongly supported by histological analysis (Figures 5–8).

### *Loss of* Krm1 *results in dramatically altered thymic architecture*

To investigate whether loss of *krm1* expression would effect proper development of the thymic architecture, K8 and K5 expressing TEC subsets were localized in thymic sections derived from fetal, neonatal and adult mice. E14.5 fetal mice showed a similar distribution of K5 and K8 cells in both *krm1* $^{+/+}$ and *krm1* $^{-/-}$ mice, with most of the cells, which were K5+, also expressing K8. K5 cells were scattered throughout the lobes in small clusters, as has been reported previously (Klug et al. 2002). The most significant difference apparent in the *krm1* $^{-/-}$ mouse was in the distribution and morphology of the K8+K5− population of TECs, which appeared more globular in morphology with less association between adjacent

◄

Figure 4. Krm1 expression in TEC subsets. Dissociated TECs from *krm1* $^{+/+}$ and *krm1* $^{-/-}$ mice were subjected to a FACS-GAL assay to detect the pattern of *Krm1* expression using the β-gal reporter. FDG loaded cells are then stained using a panel of antibodies that define subsets of cortical and medullary TECs. Panel A shows the cTEC populations defined by BP1 and EpCAM in neonatal (upper) and adult mice (lower). Panel B shows the mTEC populations defined by UEA-1 and EpCAM in neonatal (upper) and adult mice (lower). The two density plots, on the left side of each pair of panels, show the profile of the TECs obtained from *krm1* $^{+/+}$ mice (upper) and *krm1* $^{-/-}$ mice (lower) after gating on the viable $CD45^{-}$ subset. Numbers in the gates show the frequency within the $CD45^{-}$ subset. Overlay histograms, to the right of the density plots, show the frequency of *krm1* expressing cells (FDG + ) in each cTEC or mTEC subset from *krm1* $^{+/+}$ mice (red lines) and *krm1* $^{-/-}$ mice (blue lines). The *krm1* $^{+/+}$ histograms allow gating for true reporter gene expression over the endogenous β-gal background shown in the red histograms, while the frequency values show the frequency of cells in each subset of cells expressing Krm1.

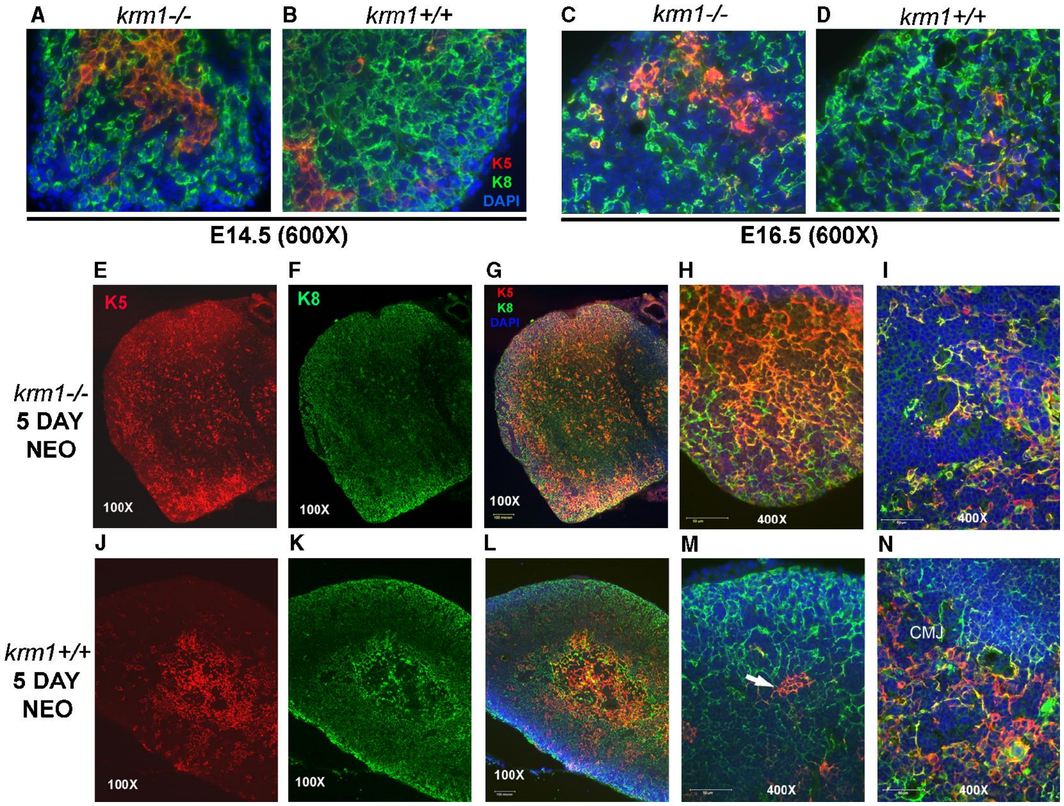

Figure 5. Keratin5 and Keratin8 expression in the thymus of fetal and 5-day neonatal Krm1$^{-/-}$ and *krm1*$^{+/+}$ littermate mice. Thymic sections stained with a mixture anti-mouse K8 (green) and anti-mouse K5 (red) followed by DAPI to detect nuclei. Figure shows a comparison of thymic architecture defined by K5 and K8 staining in E14.5 KO (A, merge) E14.5 WT (B, merge); E16.5 KO (C, merge) E16.5 WT (D, merge); 5-day neonatal KO (E) K5, (F) K8, (G) merge K5 and K8 with DAPI (mag100×), (H) 400× merge of KO cortex (note globular morphology and abundant K5 staining) and (I) 400× merge of KO medulla (note sparse epithelial distribution and abundant K5K8DP cells); 5-day neonatal WT (J) K5, (K) K8, (L) 100 × merge of K5 and K8 with DAPI, (M) 400× merge of cortex, arrow shows small patch of K8$^{-}$K5$^{+}$ cells among network of K8$^{+}$K5$^{-}$ TECs, (N) 400× CMJ showing limited K5K8 DP TECs and distinct difference in the morphology of K5 mTECs compared to K8 cTECs.

cells (Figure 5A), when compared with the *krm1*$^{+/+}$ mouse (Figure 5B). This difference in K8 cTEC morphology became more apparent at E 16.5, when gaps in the K8+cTEC network were apparent in the cortex of the *krm1*$^{-/-}$ thymus (Figure 5C) with the K8+ cells largely separated from each other and interspersed with K5+ and K5+K8+ cells (see yellow cells, Figure 5C). In contrast, in the *krm1*$^{+/+}$ thymus, the K5 cells are more centralized and the K8+ cells are interconnected in a well-organized network, making it difficult to discern individual K8+ cTECs (Figure 5D). K5+K8+ cells were still apparent in both strains.

Examination of keratin expression in 5-day neonatal animals revealed a disruption in keratin expression within TECs, and the organization of the thymic architecture, with *krm1*$^{-/-}$ animals showing no clear separation of cortical and medullary areas (Figure 5E–I) and more K5 expression, in what should be cortical areas (Figure 5E, G and H), than in central areas of the thymus. In *krm1*$^{+/+}$ animals, K5 expression was centralized (Figure 5J, L and N), surrounded by cells with typical cTEC morphology and expressing only K8 (Figure 5K–M). In *krm1*$^{+/+}$ neonatal mice, a ring of cells at the corticomedullary junction could be seen which express both K5 and K8 (see yellow cells in Figure 5L and N), while the outer cortex showed only small patches of K5-expressing TECs among a fine network of K8-expressing TECs (Figure 5M). In contrast, observation of the cortex in *krm1*$^{-/-}$ neonates revealed an abundance of K5 and double stained cells, with very limited numbers of TECs that only express K8 (Figure 5G). The morphology of the cTECs was more globular, typical of medullary cells (Figure 5H). The central area of the thymus in *krm1*$^{-/-}$ mice was sparsely populated with epithelium and contained an abundance of cells stained with both K5 and K8 (Figure 5G and I).

When we examined keratin expression in thymic sections derived from 4-week old animals, a severe disruption in the resolution of defined cortical and medullary regions of the thymus, is apparent in both *krm1*$^{-/-}$ (Figure 6A–C) and *krm1*$^{+/-}$ (Figure 6F–H) animals, when the sections are viewed at 100× magnification. Large expanses, of what should be K8$^{+}$ (green) cortical areas of the thymus, contain an

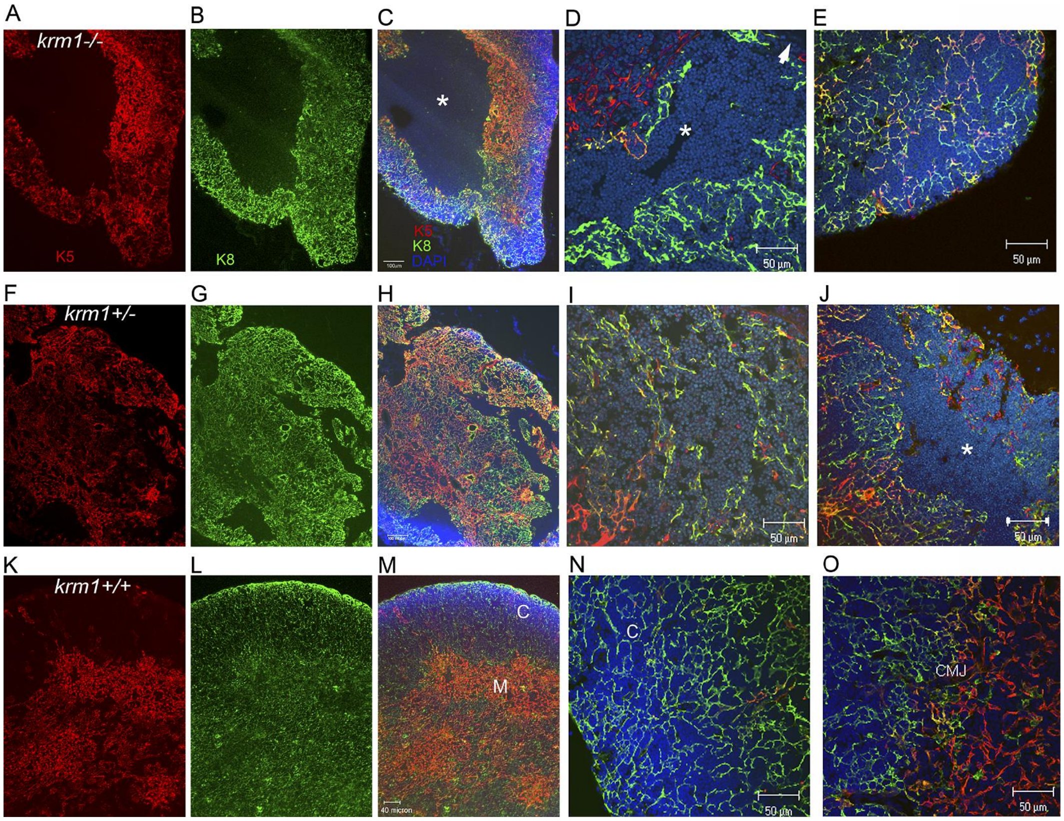

Figure 6. Keratin5 and Keratin8 expression in the thymus of 4 week-old krm1$^{+/-}$, krm1$^{-/-}$ and *krm1*$^{+/+}$ littermate mice. Cryostat sections of thymus were stained with a mixture of anti-mouse K5 (red) and anti-mouse K8 (green) followed by DAPI to detect nuclei. *Krm1*$^{-/-}$ upper row, (A) K5, (B) K8, (C) Merge with DAPI (mag100×), (D) confocal merge of an area in the cortex containing both K5+ (red) and K8+ (green) epithelial cells and a large epithelial free area (DAPI stained only ⋆) The white arrow shows the edge of the section. (E) confocal merge of cortex showing abundant K5K8 DP epithelial cells (yellow) and a loose association of the epithelial cells; *krm1*$^{+/-}$ middle row, (F) K5, (G) K8, (H) 100x merge with DAPI, (I) confocal merge of cortico-medullary junction,(lower left) to the capsule (upper right) (J) confocal merge of cortex with an epithelial free area (⋆) and abundant K5K8DP cells (yellow); *krm1*$^{+/+}$ lower row (K) K5, (L) K8, (M) 100× merge with DAPI, (N) confocal merge of outer cortex showing highly organized K8 + epithelial network with very limited K5 + TECs, (O) confocal merge of the CMJ showing limited K5K8 DP TECs. C-cortex, M-medulla, ⋆-DAPI stained epithelial free areas.

abundance of K5 (red) expressing cells (confocal, Figure 6D red cells), or cells which express both K5 and K8 (yellow, confocal Figure 6E, I and J). In addition, large areas that appear to be devoid of keratin expressing epithelial cells, and are instead only filled with lymphocytes, (see DAPI staining Figure 6C and D⋆) were present in 16 of 20 *krm1*$^{-/-}$ animals examined. These epithelial free areas often represented a large percentage of the central area of the thymus and were often flanked on one side with a region that was predominantly K5+ cells with a more globular mTEC morphology as seen in Figure 6A and C. The remaining KO mice exhibited multiple smaller epithelial free areas with K5 expressing cells often found extensively in the outer cortex, similar to the example shown in Figure 6D. *Krm1*$^{+/-}$ animals also exhibit similar but less extensive epithelial free areas (Figure 6H–J⋆). All of the *krm1*$^{-/-}$ mice showed some degree of this form of disruption, together with extensive K5K8DP TECs in both cortical and medullary areas (similar to Figure 6E). *Krm1*$^{+/+}$ animals exhibit a clear separation of cortical areas defined by the K8 and medullary areas defined by K5 (Figure 6N and O) with only limited small patches of K5 expressing TECs visible in the cortical areas (Figure 6M and N). *Krm1*$^{-/-}$ (Figure 6D and E) and *krm1*$^{+/-}$ thymi (Figure 6I and J) exhibit no clear boundary between cortical and medullary regions, with much of the cortex containing cells that have a morphology more like a subset of medullary cells (individual separated cells with fewer and thicker projections) than the typical fine stellate morphology of interconnected cTECs observed in *krm1*$^{+/+}$ animals (Figure 6N, green cTECS). Increased numbers of K5K8 DP cells were visible to varying degrees in all of the 24 adult *krm1*$^{-/-}$ and *krm1*$^{+/-}$ animals examined. The observation that similarly disrupted architecture was found in both *krm1*$^{-/-}$ and *krm1*$^{+/-}$ animals, suggests that Krm1 function is dose sensitive; loss of one allele results in aberrant development of the thymic architecture. Taken together these results clearly demonstrate that loss of *krm1* results in a deregulation of keratin expression or aberrant development of the TECs, characterized by an incomplete separation of

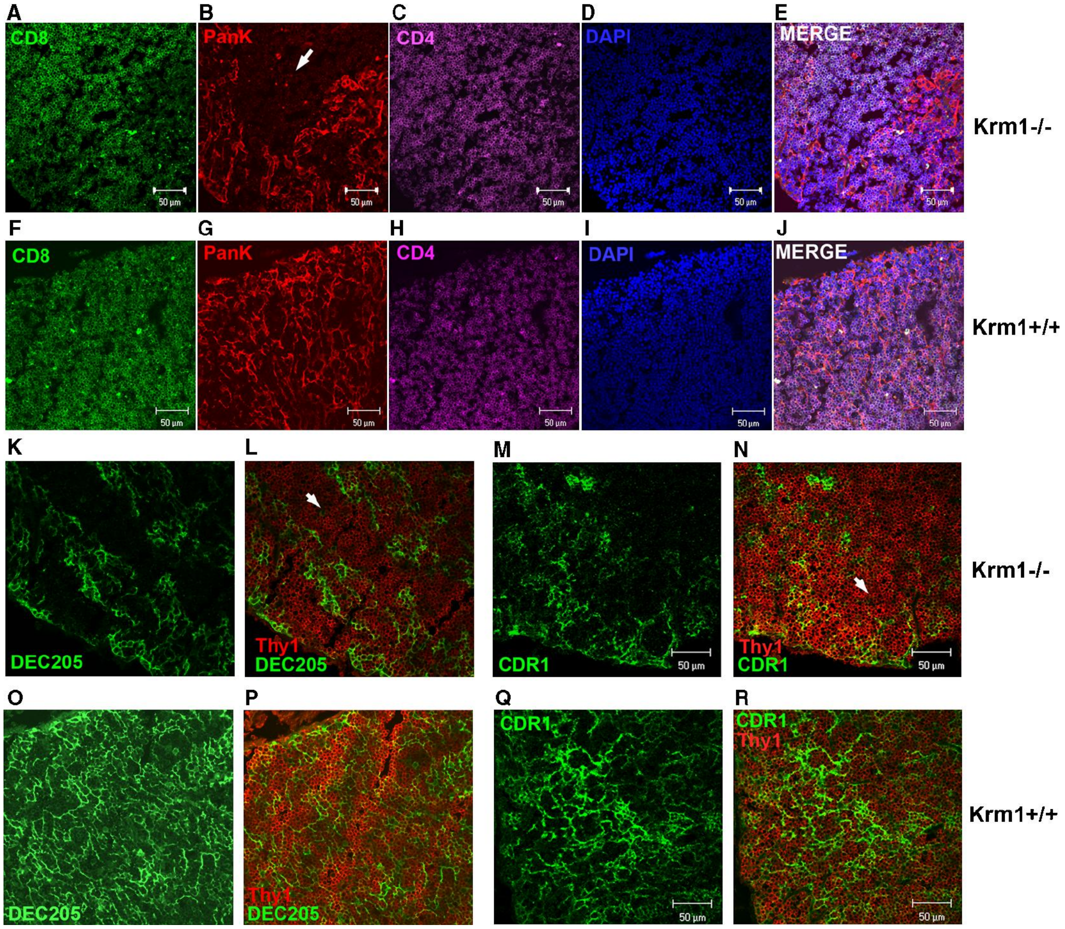

Figure 7. Confocal examination of thymic epithelial free areas and the cortical histology defined by expression of the cTEC markers CDR1 and DEC205. (A–E) *krm1* $^{-/-}$ cortical area with epithelial free region (arrow) stained with CD8 FITC (A) Pan-keratin RITC (B), CD4 APC (C), DAPI (D), and merge (E). (F–J) a similar area in a *krm1* $^{+/+}$ derived thymus stained with CD8 FITC (F), Pan-keratin RITC (G), CD4

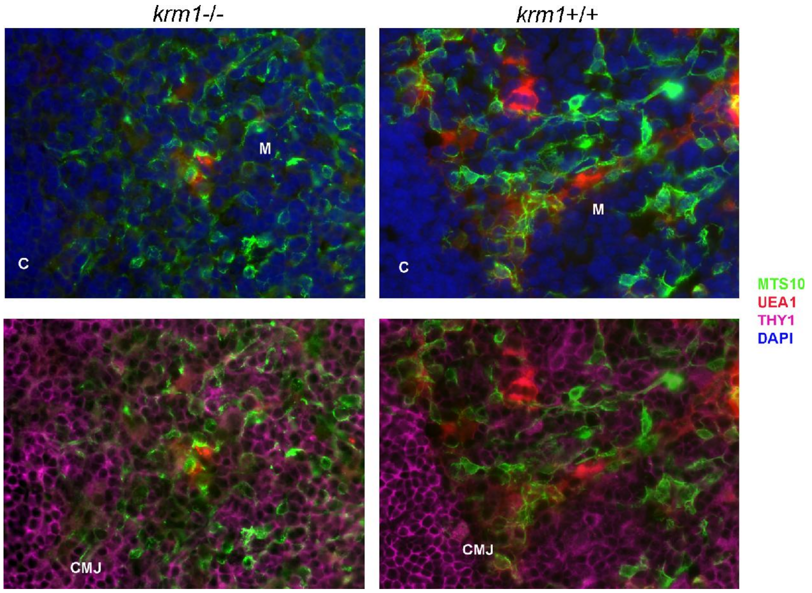

Figure 8. Analysis of medullary organization using the mTEC markers MTS10 and UEA-1. Cryostat sections of 4 week-old thymi from *Krm1* $^{-/-}$ (two left panels) and *krm1* $^{+/+}$ (two right panels) stained with rat anti-mouse MTS10 (green), UEA-1 (red) and anti-Thy 1.2 (pink). After staining, coverslips were mounted using anti-fade with DAPI (molecular probes) to stain the nuclei. Sections were photographed at 400× using a Nikon TE2000 microscope and a SPOT digital camera using identical exposures. C, cortex; M, medulla; CMJ, corticomedullary junction.

APC (H), DAPI (I) and merged (J). DEC205 FITC or CDR1 FITC staining together with Thy1 APC staining, of 8 µm cryostat sections of thymus prepared from Krm1 $^{-/-}$ mice (K–N) and *krm1* $^{+/+}$ mice (O–R). All sections were scanned using a Zeiss 510 confocal microscope.

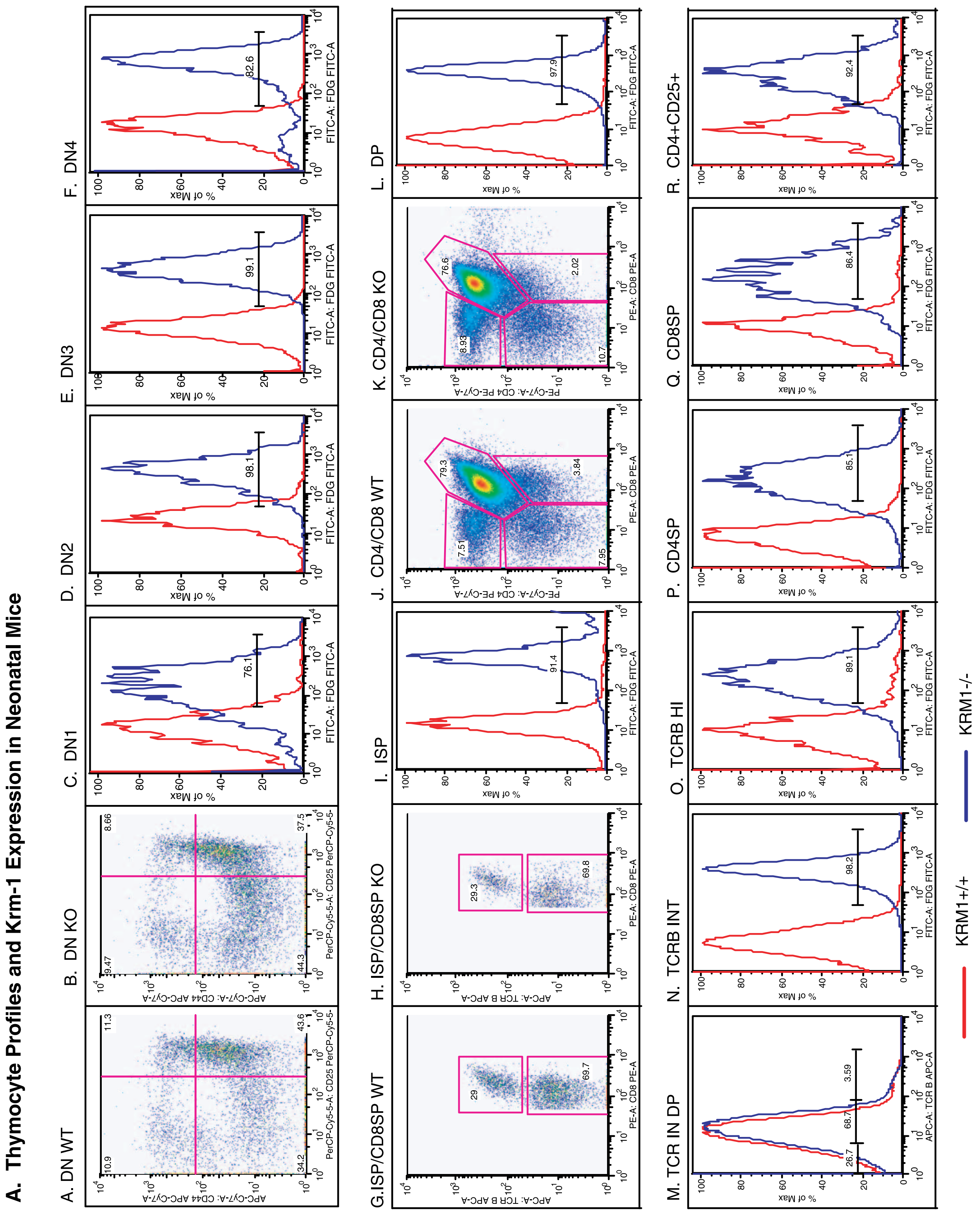

A. Thymocyte Profiles and Krm-1 Expression in Neonatal Mice

A. DN WT

B. DN KO

C. DN1

D. DN2

E. DN3

F. DN4

G.ISP/CD8SP WT

H. ISP/CD8SP KO

I. ISP

J. CD4/CD8 WT

K. CD4/CD8 KO

L. DP

M. TCR IN DP

N. TCRB INT

O. TCRB HI

P. CD4SP

Q. CD8SP

R. CD4+CD25+

KRM1+/+

KRM1-/-

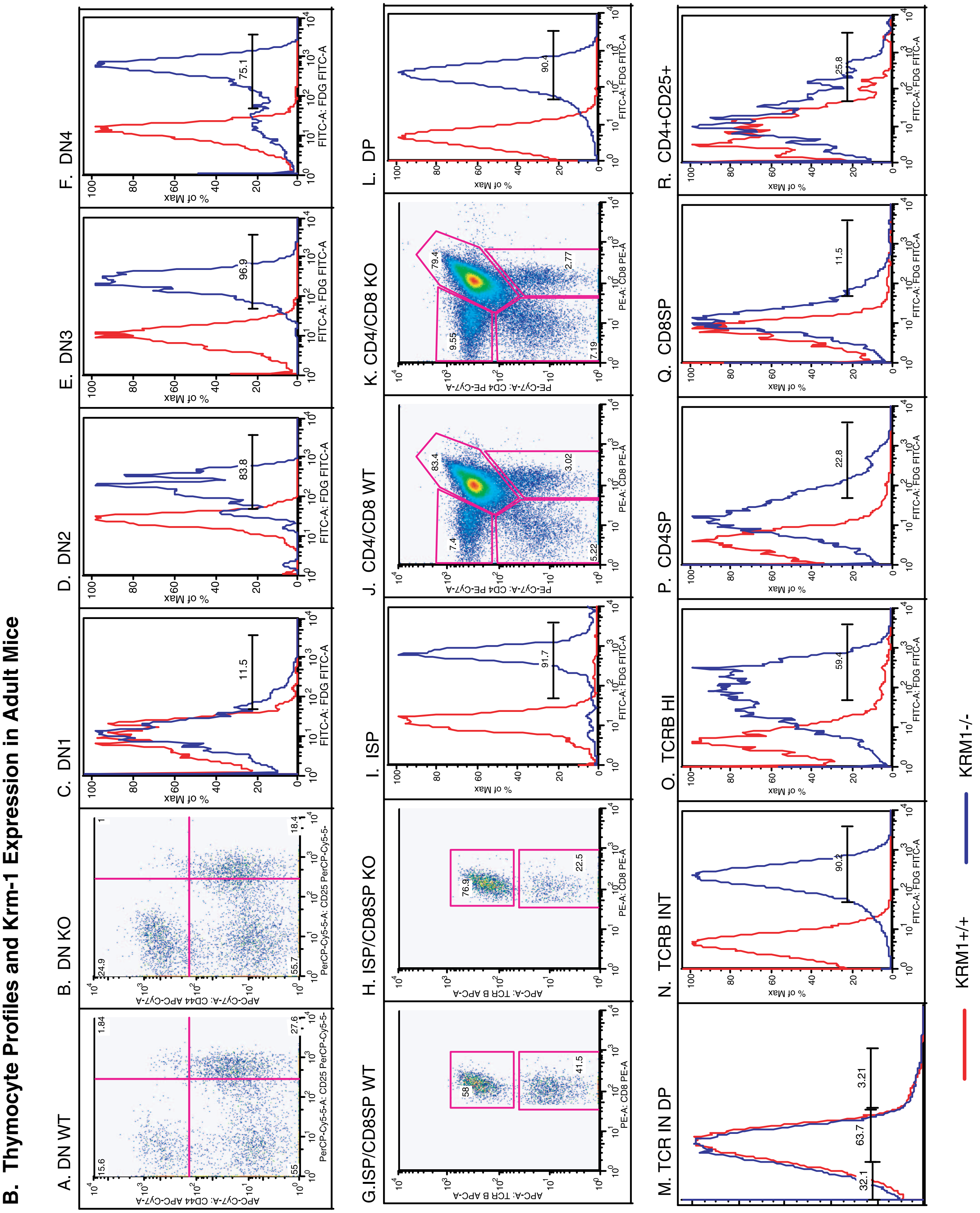

B. Thymocyte Profiles and Krm-1 Expression in Adult Mice

A. DN WT

B. DN KO

C. DN1

D. DN2

E. DN3

F. DN4

G.ISP/CD8SP WT

H. ISP/CD8SP KO

I. ISP

J. CD4/CD8 WT

K. CD4/CD8 KO

L. DP

M. TCR IN DP

N. TCRB INT

O. TCRB HI

P. CD4SP

Q. CD8SP

R. CD4+CD25+

KRM1+/+

KRM1-/-

cortical and medullary regions of the thymus or expansion of the K5/8 DP TEC progenitor population.

To confirm that the gaps in K5 and K8 staining observed in *krm1* $^{-/-}$ and *krm1* $^{+/-}$ mice are truly epithelial free regions and to identify the phenotype of the thymocytes found in these areas, we stained thymic sections with a polyclonal pan-keratin antibody, together with both CD4 and CD8 antibodies (Figure 7A–J). Pan-keratin RITC, CD8 FITC and CD4 APC staining of *krm1* $^{-/-}$ sections revealed that these K5K8 deficient areas are epithelial free, since they do not stain with the pan-keratin antibody (Figure 7B) and are filled predominantly with CD4CD8 DP thymocytes (Figure 7A–E). Similar results were obtained with *krm1* $^{+/-}$ epithelial free areas (data not shown). In contrast, staining of similar thymic cortical areas in *krm1* $^{+/+}$ mice shows an organized mesh-like epithelial network surrounding abundant DP thymocytes (Figure 7F–J). Careful examination of high magnification confocal images allowed detection of occasional SP or DN thymocytes in epithelial free areas (data not shown), however, the vast majority of the cells were DP thymocytes.

To determine if the disruption in architecture observed in *krm1* $^{-/-}$ mice was limited to keratin expression, or was also associated with other markers used to define mature cortical and medullary TECs, we examined sections of thymus from *krm1* $^{+/+}$, *krm1* $^{+/-}$ and *krm1* $^{-/-}$ animals stained with a panel antibodies including the cortical markers CDR1 and DEC205, as well as the medullary markers MTS10 and UEA-1. Staining of *krm1* $^{-/-}$ sections with both DEC205 (Figure 7K and L) and CDR1 (Figure 7M and N) revealed a loss of the perpendicular alignment of cTEC, relative to the capsule, that is characteristic of the *krm1* $^{+/+}$ animals (Figure 7O–R). Instead the CDR1 and DEC205 expressing cTECs appear to cluster together in clumps with large gaps that appeared to be epithelial free and filled with thymocytes, indicated by the Thy1 staining which fills these areas (Figure 7L and N, arrows). Analysis of mTECs showed that the intensity of staining for both MTS10 (green) and UEA-1 (red) was reduced in *krm1* $^{-/-}$ sections (Figure 8, left 2 panels) when compared to the *krm1* $^{+/+}$ sections (Figure 8, right panels). In addition, there were fewer UEA-1$^{+}$ cells and a more globular morphology for both the UEA-1$^{+}$ and MTS10 + cells in the *krm1* $^{-/-}$ sections. Analysis of both DAPI (Figure 8, upper panels) and Thy1.2 staining (Figure 8, lower panels), shows that the division between cortex and medulla is less defined in the *krm1* $^{-/-}$ thymus, when compared to *krm1*$^{+/+}$ animals, with more thymocytes infiltrating the medullary areas, defined by MTS10 and UEA-1 staining, in the *krm1* $^{-/-}$ thymus. MTS10 and UEA-1 remain distributed in the central areas of the thymus (Figure 8), similar to *krm1* $^{+/+}$ animals; however, the overall staining level for both markers on individual cells is reduced. Staining with UEA-1 on different KO mice, demonstrates that the frequency of UEA-1 binding cells is highly variable, with some mice exhibiting UEA-1 staining patterns that were nearly identical to *krm1* $^{+/+}$ controls and others showing almost no UEA-1$^{+}$ cells, similar to the example shown in Figure 8. This variation was never observed in *krm1* $^{+/+}$ animals. The decrease in TECs, observed in the histology, was also reflected in a decrease in frequency of both UEA1+EpCAM+ medullary and the BP1+EpCAM+, cortical epithelial types when FACS analysis of dissociated thymic lobes was performed (see Figure 4, profiles of *krm1* $^{+/+}$ vs. *krm1* $^{-/-}$ animals). CDR1 and BP1 stain the same population of cTECs.

### Krm1 *is differentially expressed in thymocyte subsets, however loss of* krm1 *has no effect on thymocyte number or frequency*

The frequency of thymocyte subsets in both *krm1* $^{+/+}$ and *krm1* $^{-/-}$ animals was determined for both neonatal (Figure 9A, scatter plots A and B) and adult mice (Figure 9B, scatter plots A and B) using multicolor FACS analysis to determine if loss of *krm1* effects T cell development. No significant differences in the frequency of the various thymocyte subsets from DN1 to SP were observed after analysis of 12 adult mice and 8 neonatal mice of each genotype (*krm1* $^{+/+}$, *krm1* $^{+/-}$ and *krm1* $^{-/-}$). A representative comparison of *krm1* $^{+/+}$ and *krm1* $^{-/-}$ profiles observed together with the frequency of *krm1* expressing cells in each subset is provided for both neonatal and adult animals. Analysis of krm1 expression using the FACS-gal assay in neonatal thymocytes (Figure 9A, histograms, blue lines) revealed that *krm1* was expressed in all subsets, with only slightly reduced frequency in the DN1 (76%) and SP subsets (85%) (slight reductions begin at the

Figure 9. (A) and (B) Analysis of T cell development and *krm1* expression in thymocyte subsets. A FACS based comparison of the frequency of thymocyte subsets in neonatal (9A) and adult (9B) Krm1KO and WT mice as well as the expression of *krm1* in each thymocyte subset, based on a FACS-GAL assay. Each panel of dot plots and histograms is organized in the order that the cells appear in development. For both Figure 9A and B the frequency of DN subsets is shown in panel (A) for WT mice and panel (B) for KO mice. The overlay histograms show krm1 expression (FDG+) in the DN subsets panel (C) DN1, (D) DN2, (E) DN3, and (F) DN4. Figure 9A and B, panels G–H, show a comparison of the frequency of ISPs, followed by an overlay (panel I) showing Krm1 frequency in the ISP subset. Panels J and K, allow comparison of the CD4/CD8 profile in *krm1* WT (J) and *krm1* KO mice (K). Panel L shows the frequency of Krm1+ cells in the DP subset, while panel M shows the frequency of TCRβ expressing cells in the DP subset. The remaining overlay histograms show the frequency of Krm1+ cells within the DP TCRβ$^{int}$ (N), DP TCRβ$^{hi}$ (O), CD4SP (P), CD8SP (Q) and CD4$^{+}$CD25$^{+}$ subset (R).

DPTCR$^{hi}$ stage). Two distinct populations were apparent in the DN4 population, however the DN4 population may also contain non-T cell subsets since NK, B and DCs were not excluded from the analysis. A similar pattern of *krm1* expression was observed in adult thymocytes (Figure 9B, histograms); however, only 11% of the DN1 population was *krm1*$^{+}$ (C), suggesting that in adults, *krm1* expression is initiated at the DN1–DN2 transition. High expression continues until the DPTCR$^{int}$ to DPTCR$^{hi}$ transition (N, O), where the frequency of *krm1*$^{+}$ cells drops from 90 to 59%, respectively. This suggests that the decline in krm1 is associated with positive selection. Panel M shows the gating used for TCR-expression in the DP population. Although the variation in total cell numbers was greater for krm1$^{+/-}$ and *krm1*$^{-/-}$ animals, no difference in the mean total thymocyte numbers, recovered from 5 adult and 5 neonatal mice of each genotype, was observed (data not shown). No difference in the mean frequency of individual thymocyte subsets, obtained from a comparison of 7 adult mice of each genotype, was found (data not shown).

### Discussion

This study makes two important contributions to our understanding of the signaling mechanisms, which regulate TEC differentiation during thymic organogenesis. First it demonstrates that Krm1, a key negative regulator of the canonical Wnt signaling pathway, is expressed in a high frequency of both immature thymocyte subsets (Figure 9A and B) as well as the TEC components of the thymic stroma in neonatal mice (Figures 1B and 4). In adult thymocytes, initiation of *krm1* expression begins at the DN1–DN2 transition and is turned off at the DP TCR$^{int}$–DP TCR$^{hi}$ transition (Figure 9B). TEC expression of *krm1* is strongly down regulated in adult mice, after the expansion stage of the stroma is complete (Figures 3F and 4). Our results are consistent with the results of Pongracz et al., demonstrating that the receptors responsible for initiating the canonical Wnt signaling cascade, LRP-6 and Fz, as well as Wnt 1 and Wnt 4 are expressed in TECs. We extend their work by demonstrating that the recently identified canonical Wnt signaling antagonist, *krm1*, is also expressed in TECs, together with the soluble regulators Dkk2 and Dkk3 (Figure 1C). Second, using a newly developed *krm1* KO mouse, we demonstrate that loss of *krm1* results in a 2-fold increase in canonical Wnt signaling within the TEC components of the thymus (Figure 2). Using this krm1 KO mouse model, we demonstrate that, while loss of *krm1* has no apparent effect on the frequency of specific thymocyte subsets or the total number of thymocytes in the post-natal thymus (Figure 9), it results in aberrant development of the thymic architecture (Figures 5–8). These differences include a persistence or expansion of a subset of TECs, which express both K5 and K8, limited development of 3D-organized K8$^{+}$K5$^{-}$ cTECS (Figures 5–6) and extensive epithelial free areas filled with DP thymocytes. In addition, we observe a general decrease in the frequency of TECs of both mature cortical and medullary phenotypes in *krm1*$^{-/-}$ animals, when compared with *krm1*$^{+/+}$ controls, suggesting a reduced expansion of the epithelial components during development of the thymus or a maintenance of K5K8DP immature subsets (Figure 4).

The normal post-natal thymus consists of a highly organized network of stromal elements with specific thymocyte subsets localized within discrete environments or layers, as they progress through their development (Lind et al. 2001). This organization suggests, that these microenvironments provide the specific signals necessary to support the development or expansion of specific thymocyte subsets. Very little is known, however, about the signals provided by these microenvironments or the molecular mediators, which regulate the movement of specific thymocyte subsets within these locations in the stroma. CXCR4 signaling, and production of its ligand, CXCL12 by cTECS, has been shown to be important in ensuring that immature thymocytes reside within the cortex (Plotkin et al. 2003), while CCR7 (produced by TECS) has been implicated in controlling thymocyte migrations from the CMJ to the cortex (Misslitz et al. 2004) and then at later stages from the cortex to the medulla (Ueno et al. 2004). In addition to chemokines, cell-cell interactions between thymocytes and stromal components, mediated by adhesion molecules and integrins, also appear to be important to thymocyte migration and differentiation (Prockop et al. 2002). In light of the mounting evidence to support the idea that proper organization of stromal microenvironments is critical for thymocyte recruitment, expansion and migration, it is surprising that postnatal T cell development appears normal in the disorganized stroma of the Krm1 KO mouse. While the TEC components are disorganized, and the individual TECs have a different morphology, FACs analysis shows that all of the mature populations appear to be present (though in reduced numbers). This may suggest that the fine structure of the thymus is less critical to the migration and development of thymocytes, than the mere presence of distinct epithelial subsets. Alternatively, normal islands of proper organization may be sufficient to regulate thymocyte migration and development in the Krm1 KO mouse.

Signaling pathways initiated by secreted Wnt glycoproteins have been shown to be important to proper development of a number of organs, including the kidney (Perantoni 2003), mammary gland (Hatsell et al. 2003), uterus (Mericskay et al. 2004), pancreas (Heller et al. 2002), lacrimal gland and lung

(Dean et al. 2005). Like the thymus, proper development of these organs involves complex interactions between developing epithelium and associated mesenchyme derived tissues with secreted morphogens like BMPs, SHHs, FGFs and Wnts appearing to regulate the expansion, migration, gene expression profile and differentiation of both the mesenchyme and epithelial components. During the branching morphogenesis of the lung and lacrimal gland, over-expression of Wnts, stimulation of canonical Wnt signaling with lithium chloride and conditionally over-expressing β-catenin in the lacrimal gland epithelium all resulted in a decrease in branching morphogenesis (Dean et al. 2005). In this system, Wnt signaling functions as a negative regulator of epithelial cell proliferation and appears to act by directly decreasing *Fgf10* expression in the mesenchyme or by directly counteracting the proliferative signals provided by BMPs produced in the mesenchyme. The development of the 3D mesh-like network of TECs in the thymus increases the surface area of the epithelium and maximizes contact of the TECs with subsets of developing thymocytes, while allowing the thymocytes to freely migrate through the various regions of the thymus. This is reminiscent of the goals of branching morphogenesis in organs like the lung, where a mesh-like epithelial network maximizes surface area for gas exchange. Subsets of thymic epithelium and lung epithelium have recently been shown to share common morphological features and express of many genes previously thought to be restricted to either lung or thymus (Dooley et al. 2005a). These cells share a phenotypic resemblance to the $K5^+K8^+$ TEC progenitors. In nude mice, which lack functional FoxN1, thymic epithelium takes on phenotypic properties and tissue organization, which strongly resembles respiratory epithelium (Dooley et al. 2005b).

Alterations in BMP or Fgf signaling in the thymus result in TEC phenotypes, which are similar to the phenotype observed in Krm1 KO mice. Inhibition of BMP signaling in the thymus, through epithelial directed transgenic expression of the BMP antagonist Noggin, under the control of a Foxn1 promoter in mice, leads to dysplastic thymic lobes of dramatically reduced size, that fail to migrate caudally and ventrally to their position above the heart (Bleul and Boehm 2005). In these mice, control of BMP2 and 4 expression in both the epithelial and mesenchyme cells is lost, resulting in defects in development of the TECs without effecting T cell development. These mice develop epithelial cysts of abnormal epithelium, however, the thymus also contains disorganized regions of both cortical and medullary TECs, as defined by exclusive expression of K8 and K5, respectively. Both Fgf7 and Fgf10 are expressed by the mesenchyme surrounding the thymus from E13.5, while at the same time the receptor for these growth factors, FgfR2-IIIb, is expressed by TECs. FgfR2-IIIb deficient mice show a severe block in thymic epithelial proliferation starting between E12 and E13, which results in drastically reduced thymic size and an abundance of TECs that retain the $K5^+K8^+$ phenotype (Revest et al. 2001). Mice deficient in Fgf10 show a similar but less severe thymic hypoplasia (Ohuchi 2000; Revest et al. 2001).

In this study, we demonstrate that *krm*1 is highly expressed in the epithelial components of the neonatal thymus (Figures 1, 3 and 4), together with all the necessary components of the canonical Wnt signaling pathway, as well as the soluble Wnt regulators Dkk2 and Dkk3. Krm1 functions as a negative regulator of the canonical Wnt signaling pathway by interacting with DKK and sequestering LRP-6, the co-receptor for Wnts (Mao et al. 2001; Davidson et al. 2002; Mao et al. 2002; Mao and Niehrs 2003). The *krm1* knockout mice then represent a model in which a key regulator of canonical Wnt signaling is lost, resulting in excessive Wnt signaling in cells like TECs. Using a TOPFlash assay, we confirm that primary TEC lines, derived from the *krm1* KO mouse, exhibit a 2-fold increase in endogenous Wnt signaling (Figure 2). This suggests that krm1 plays a significant role in regulating canonical Wnt signaling in the thymus. Kremen is the only membrane bound inhibitor known, while a total of eleven soluble factors, which interfere with the binding of Wnt to its receptor complex, have been identified (Kawano and Kypta 2003). A balance of soluble Wnts stimulating canonical Wnt signaling in target cells and Wnt inhibitory proteins including Dkk and Krm on the same targets, has been shown to be critical in anterior posterior CNS patterning during early embryogenesis (Davidson et al. 2002). In the absence of *krm1*, we observed dramatic alterations of TEC differentiation and organization including a lack of defined cortical and medullary areas and an abundance of $K5^+K8^+$ TECs, not normally abundant in the *krm1* $^{+/+}$ thymus at comparable developmental stages (Figure 5 and 6). The observed differences in TEC development may be the result of a disruption in the balance between BMP and/or FGF driven proliferation or differentiation signals derived from the mesenchyme and Wnt driven signaling in the TECs, similar to the BMP/Fgf and Wnt interaction reported for lung and lacrimal gland development (Dean et al. 2005). Future studies should address whether loss of Krm1 expression and the associated increase in canonical Wnt signaling contribute to alterations in BMP or FGF regulation or expression in the thymus.

Examination of the TECs in nude mice has shown that the early phases of thymic organogenesis occur independently of interactions with hematopoietic-derived cells (Nehls et al. 1996). Lympho-stromal interactions (crosstalk) influence the normal development of T cells as well as thymic architecture during later phases of thymic development (Shores et al. 1991; van Ewijk et al. 2000). Down regulation of K5 in the thymic cortex has been reported to depend on crosstalk between developing thymocytes and TEC precursors expressing both K5 and K8 (Klug et al. 1998; 2000). In RAG-1 deficient mice, where T cell development progresses to the $CD44^{-}CD25^{+}$ DN3 stage the cortex is well organized with K8+K5− TECs, however in human CD3ε transgenic RAG-$2\gamma c^{-/-}$ thymi with earlier blocks in development the epithelium is poorly organized with abundant $K5^{+}K8^{+}$ TECs, similar to the phenotype we observed in $Krm1^{-/-}$ mice. Medullary organization was abnormal in $TCR\alpha^{-/-}$ mice, suggesting that development of mTECs was dependent on direct interactions or soluble factors provided by $\alpha/\beta TCR^{+}$ thymocytes (Palmer et al. 1993). $Krm1^{-/-}$ mice exhibit normal T cell development with normal thymocyte numbers (Figure 9). Given this observation, the differences in thymic architecture observed in *krm1* $^{-/-}$ mice do not appear to be related to an absence of the necessary thymocyte subsets responsible for crosstalk. An alternative explanation might be that Wnt signaling plays a role in the crosstalk that occurs between developing thymocytes and the TEC progenitors, which give rise to the mature $K8^{+}K5^{-}$ cTEC network, as well as the production of mature mTEC subsets. Even though the appropriate Wnt expressing thymocyte subsets are present in the *krm1* $^{-/-}$ thymus, in the absence of the key canonical Wnt signaling regulator *krm1*, excessive Wnt signaling might disrupt the signals necessary to drive or regulate TEC differentiation. A recent study has suggested that development of functional mature (K5+K8− and K5−K8+) TECs occur independently of lymphostromal interactions in fetal mice, but thymocytes are needed to maintain an organized architecture in the post natal thymus (Jenkinson et al. 2005). Our analysis of K5 and K8 expression in the *krm1* $^{-/-}$ fetal mouse suggests that while keratin expression and the distribution of TECs is similar in both WT and KO E14.5 mice, defects in the organization of $K8^{+}$ cTECs are already apparent (Figure 5A and B). At E16.5 the globular morphology of K8+ cTECs and an increase in K5+ and K8K5 DP cells in cortical areas of the *krm1* $^{-/-}$ mice is more apparent (Figure 5C and D). In addition, we begin to see the gaps and disorganization in the cTEC network that characterizes the cortex of *krm1* $^{-/-}$ adult mice (Figures 6–7). K8+ and K5+ cells appear to develop in the absence of Wnt signaling inhibition by Krm1, however, K5 medullary cells are not centralized and K8K5 DP cells either expand in number or at least persist in the *krm1* $^{-/-}$ mouse into adulthood. Expression of both K5 and K8 by the TECs in *krm1* $^{-/-}$ and *krm1* $^{+/-}$ animals is a characteristic that has been attributed to more immature TECs. K8+K5+ TECs first appear during fetal development at E12.5 and persist until E17.5, when well defined cortical and medullary boundaries appear (Klug et al. 2002). Alternatively, expression of K5 together with K8 was attributed to a small population of TEC progenitors that persist at the cortico-medullary junction in adults. When isolated from immature mice these K5+K8+ cells can give rise to a complete thymus, when transferred under the kidney capsule in nude mice (Gill et al. 2002). Regulation of Wnt signaling, possibly through crosstalk, may be important in maintaining the TEC organization, rather than initial development of mature TEC subsets. Anderson et al. (2006), has suggested that thymocytes may provide signals required for the maintenance of a properly formed cortex and medulla. In the absence of these signals, the only cells that survive are the immature K8K5 DP cells, which then expand in number. Alternatively, in the absence of signals derived from thymocytes and a proper 3D organization, the epithelial components may change their keratin expression pattern, effectively undergoing de-differentiation (Anderson et al. 2006). The thymic architecture defects we observed in Krm1KO mice are not due to a lack of normal thymocyte subsets, since the frequency and number of distinct thymocyte subsets appears normal in *krm1* $^{-/-}$ mice (Figure 9 and data not shown). This might suggest that one of the important crosstalk signals that thymocytes provide is Wnt inhibitory signals. In our model, in the absence of the membrane bound Wnt inhibitor Krm1, excessive Wnt signaling in the TECs mimics the lack of crosstalk signals responsible for the architecture defects observed in mice with T cell developmental blocks. The presence of normal thymocyte frequencies, taken together with the fact that we observe a 2-fold increase in endogenous canonical Wnt signaling within KO derived TECs (Figure 2), in the absence of thymocytes, would suggest that the defect we are observing may be TEC autonomous or dependent on epithelial/mesenchyme interactions, rather than thymocyte/TEC interactions. This question will be important to address in future studies of Wnt signaling in thymic organogenesis using bone marrow chimeric animals. Whether *krm1* regulation of Wnt signaling is important for maintenance of mature TECs or differentiation from immature subsets, the results of this study clearly demonstrate an important role for regulation of canonical Wnt signaling by *krm1*, in the proper organization of the thymic architecture.

### Acknowledgements

This work was supported by NIH Grant Number 5G12 RR03060-21 from the NCRR, NIH/NIGMS # SO6 GM 008168, NIH-NCI U56 CA96299-01 and PSCCUNY Award 67381-0036.

We would like to thank Dr Marc Tessier-Lavigne, for the use of his reagents and facilities at Stanford

University, for the production of the Krm1 knockout mice. Dr Christof Niehrs, Division of Molecular Embryology, Deutsches Krebsforschungszentrum and Dr Toshikazu Nakamura, Graduate School of Medicine, Osaka University, kindly provided the Krm1 plasmids, used to generate the probes for *in situ* hybridization. We sincerely thank Dr Howard Petrie, Dr Christine Li and Dr Jerry Guyden for their helpful comments during the preparation of this manuscript and Mr. Jeffrey Walker for his technical assistance with FACS analysis.

The TROMA-1 antibody developed by Philippe Brulet and Rolf Kemler was obtained from the Developmental Studies Hybridoma Bank developed under the auspices of the NICHD and maintained by the University of Iowa, Department of Biological Sciences, Iowa City, IA 52242.

### References

Anderson MS, Venanzi ES, Klein L, Chen Z, Berzins SP, Turley SJ, von Boehmer H, Bronson R, Dierich A, Benoist C, Mathis D. 2002. Projection of an immunological self shadow within the thymus by the aire protein. Science 298:1395–1401.

Anderson G, Jenkinson WE, Jones T, Parnell SM, Kinsella FA, White AJ, Pongrac'z JE, Rossi SW, Jenkinson EJ. 2006. Establishment and functioning of intrathymic microenvironments. Immunol Rev 209:10–27.

Balciunaite G, Keller MP, Balciunaite E, Piali L, Zuklys S, Mathieu YD, Gill J, Boyd R, Sussman DJ, Hollander GA. 2002. Wnt glycoproteins regulate the expression of *FoxN1*, the gene defective in nude mice. Nat Immunol 3:1102–1108.

Barclay AN, Mayrhofer G. 1981. Bone marrow origin of Ia-positive cells in the medulla rat thymus. J Exp Med 153:1666–1671.

Bennett AR, Farley A, Blair NF, Gordon J, Sharp L, Blackburn CC. 2002. Identification and characterization of thymic epithelial progenitor cells. Immunity 16:803–814.

Blackburn CC, Augustine CL, Li R, Harvey RP, Malin MA, Boyd RL, Miller JF, Morahan G. 1996. The *nu* gene acts cell-autonomously and is required for differentiation of thymic epithelial progenitors. Proc Natl Acad Sci USA 93:5742–5746.

Bleul CC, Boehm T. 2005. BMP Signaling is required for normal thymus development. J Immunol 175:5213–5221.

Davidson G, Mao B, del Barco Barrantes I, Niehrs C. 2002. Kremen proteins interact with Dickkopf1 to regulate anteroposterior CNS patterning. Development 129:5587–5596.

Dean CH, Miller LA, Smith AN, Dufort D, Lang RA, Niswander LA. 2005. Canonical Wnt signaling negatively regulates branching morphogenesis of the lung and lacrimal gland. Dev Biol 286:270–286.

Dooley J, Erickson M, Farr AG. 2005a. An organized medullary epithelial structure in the normal thymus expresses molecules of respiratory epithelium and resembles the epithelial thymic rudiment of nude mice. J Immunol 175:4331–4337.

Dooley J, Erickson M, Roelink H, Farr AG. 2005b. Nude thymic rudiment lacking functional FoxN1 resembles respiratory epithelium. Dev Dyn 233:1605–1612.

van Ewijk W, Wang B, Hollander G, Kawamoto H, Spanopoulou E, Itoi M, Amagai T, Jiang YF, Germeraad WT, Chen WF, Katsura Y. 1999. Thymic microenvironments, 3D vs. 2D? Semin Immunol 11:57–64.

van Ewijk W, Hollander G, Terhorst C, Wang B. 2000. Stepwise development of thymic microenvironments *in vivo* is regulated by thymocyte subsets. Development 127:1583–1591.

Ferrick DA, Sambhara SR, Ballhausen W, Iwamoto A, Pircher H, Walker CL, Yokoyama WM, Miller RG, Mak TW. 1989. T cell function and expression are dramatically altered in T cell receptor V gamma 1.1J gamma 4C gamma 4 transgenic mice. Cell 57:483–492.

Friedel RH, Plump A, Lu X, Spilker K, Jolicoeur C, Wong K, Venkatesh TR, Yaron A, Hynes M, Chen B, Okada A, McConnell SK, Rayburn H, Tessier-Lavigne M. 2005. Gene targeting using a promoterless gene trap vector ("targeted trapping") is an efficient method to mutate a large fraction of genes. Proc Natl Acad Sci USA 102:13188–13193.

Gabor MJ, Godfrey DI, Scollay R. 1997. Recent thymic emigrants are distinct from most medullary thymocytes. Eur J Immunol 27:2010–2015.

Gill J, Malin M, Hollander GA, Boyd R. 2002. Generation of a complete thymic microenvironment by MTS24(+) thymic epithelial cells. Nat Immunol 3:635–642.

Gotter J, Brors B, Hergenhahn M, Kyewski B. 2004. Medullary epithelial cells of the human thymus express a highly diverse selection of tissue-specific genes colocalized in chromosomal clusters. J Exp Med 199:155–166.

Gray DH, Chidgey AP, Boyd RL. 2002. Analysis of thymic stromal cell populations using flow cytometry. J Immunol Methods 260:15–28.

Hatsell S, Rowlands T, Hiremath M, Cowin P. 2003. Beta-catenin and Tcfs in mammary development and cancer. J Mammary Gland Biol Neoplasia 8:145–158.

Hattori N, Kawamoto H, Fujimoto S, Kuno K, Katsura Y. 1996a. Involvement of transcription factors TCF-1 and GATA-3 in the initiation of the earliest step of T cell development in the thymus. J Exp Med 184:1137–1147.

Hattori N, Kawamoto H, Katsura Y. 1996b. Isolation of the most immature population of murine fetal thymocytes that includes progenitors capable of generating T, B, and myeloid cells. J Exp Med 184:1901–1908.

Heller RS, Dichmann DS, Jensen J, Miller C, Wong G, Madsen OD, Serup P. 2002. Expression patterns of Wnts, Frizzleds, sFRPs, and misexpression in transgenic mice suggesting a role for Wnts in pancreas and foregut pattern formation. Dev Dyn 225:260–270.

Ioannidis V, Beermann F, Clevers H, Held W. 2001. The beta-catenin—TCF-1 pathway ensures CD4(+)CD8(+) thymocyte survival. Nat Immunol 2:691–697.

Jenkinson WE, Rossi SW, Jenkinson EJ, Anderson G. 2005. Development of functional thymic epithelial cells occurs independently of lymphostromal interactions. Mech Dev 122: 1294–1299.

Kawano Y, Kypta R. 2003. Secreted antagonists of the Wnt signalling pathway. J Cell Sci 116:2627–2634.

Klug DB, Carter C, Crouch E, Roop D, Conti CJ, Richie ER. 1998. Interdependence of cortical thymic epithelial cell differentiation and T-lineage commitment. Proc Natl Acad Sci USA 95:11822–11827.

Klug DB, Crouch E, Carter C, Coghlan L, Conti CJ, Richie ER. 2000. Transgenic expression of cyclin D1 in thymic epithelial precursors promotes epithelial and T cell development. J Immunol 164:1881–1888.

Klug DB, Carter C, Gimenez-Conti IB, Richie ER. 2002. Cutting edge: Thymocyte-independent and thymocyte-dependent phases of epithelial patterning in the fetal thymus. J Immunol 169:2842–2845.

Kyewski B, Derbinski J. 2004. Self-representation in the thymus: An extended view. Nat Rev Immunol 4:688–698.

Leyns L, Bouwmeester T, Kim SH, Piccolo S, De Robertis EM. 1997. Frzb-1 is a secreted antagonist of Wnt signaling expressed in the Spemann organizer. Cell 88:747–756.

Lind EF, Prockop SE, Porritt HE, Petrie HT. 2001. Mapping precursor movement through the postnatal thymus reveals specific microenvironments supporting defined stages of early lymphoid development. J Exp Med 194:127–134.

Li L, Mao J, Sun L, Liu W, Wu D. 2002. Second cysteine-rich domain of Dickkopf-2 activates canonical Wnt signaling pathway via LRP-6 independently of dishevelled. J Biol Chem 277:5977–5981.

Manley NR. 2000. Thymus organogenesis and molecular mechanisms of thymic epithelial cell differentiation. Semin Immunol 12:421–428.

Mao B, Niehrs C. 2003. Kremen2 modulates Dickkopf2 activity during Wnt/LRP6 signaling. Gene 302:179–183.

Mao B, Wu W, Li Y, Hoppe D, Stannek P, Glinka A, Niehrs C. 2001. LDL-receptor-related protein 6 is a receptor for Dickkopf proteins. Nature 411:321–325.

Mao B, Wu W, Davidson G, Marhold J, Li M, Mechler BM, Delius H, Hoppe D, Stannek P, Walter C, Glinka A, Niehrs C. 2002. Kremen proteins are Dickkopf receptors that regulate Wnt/beta-catenin signalling. Nature 417:664–667.

Mericskay M, Kitajewski J, Sassoon D. 2004. Wnt5a is required for proper epithelial–mesenchymal interactions in the uterus. Development 131:2061–2072.

Miller JR. 2002. The Wnts. Genome Biol 3, REVIEWS3001.

Misslitz A, Pabst O, Hintzen G, Ohl L, Kremmer E, Petrie HT, Forster R. 2004. Thymic T cell development and progenitor localization depend on CCR7. J Exp Med 200:481–491.

Muller SM, Terszowski G, Blum C, Haller C, Anquez V, Kuschert S, Carmeliet P, Augustin HG, Rodewald HR. 2005. Gene targeting of VEGF-A in thymus epithelium disrupts thymus blood vessel architecture. Proc Natl Acad Sci USA 102:10587–10592.

Nehls M, Kyewski B, Messerle M, Waldschutz R, Schuddekopf K, Smith AJ, Boehm T. 1996. Two genetically separable steps in the differentiation of thymic epithelium. Science 272:886–889.

Niehrs C. 1999. Head in the Wnt: The molecular nature of Spemann's head organizer. Trends Genet 15:314–319.

Ohuchi. 2000. FGF10 acts as a major ligand for FGF receptor 2 IIIb in mouse multi-organ development. Biochem Biophys Res Commun 277:643.

Palmer DB, Viney JL, Ritter MA, Hayday AC, Owen MJ. 1993. Expression of the alpha beta T-cell receptor is necessary for the generation of the thymic medulla. Dev Immunol 3:175–179.

Perantoni AO. 2003. Renal development: Perspectives on a Wnt-dependent process. Semin Cell Dev Biol 14:201–208.

Plotkin J, Prockop SE, Lepique A, Petrie HT. 2003. Critical role for CXCR4 signaling in progenitor localization and T cell differentiation in the postnatal thymus. J Immunol 171: 4521–4527.

Pongracz J, Hare K, Harman B, Anderson G, Jenkinson EJ. 2003. Thymic epithelial cells provide Wnt signals to developing thymocytes. Eur J Immunol 33:1949–1956.

Prockop SE, Palencia S, Ryan CM, Gordon K, Gray D, Petrie HT. 2002. Stromal cells provide the matrix for migration of early lymphoid progenitors through the thymic cortex. J Immunol 169:4354–4361.

Revest JM, Suniara RK, Kerr K, Owen JJ, Dickson C. 2001. Development of the thymus requires signaling through the fibroblast growth factor receptor R2-IIIb. J Immunol 167: 1954–1961.

Savage PA, Davis MM. 2001. A kinetic window constricts the T cell receptor repertoire in the thymus. Immunity 14:243–252.

Schilham MW, Wilson A, Moerer P, Benaissa-Trouw BJ, Cumano A, Clevers HC. 1998. Critical involvement of Tcf-1 in expansion of thymocytes. J Immunol 161:3984–3991.

Shores EW, Van Ewijk W, Singer A. 1991. Disorganization and restoration of thymic medullary epithelial cells in T cell receptor-negative scid mice: Evidence that receptor-bearing lymphocytes influence maturation of the thymic microenvironment. Eur J Immunol 21:1657–1661.

Staal FJ, Meeldijk J, Moerer P, Jay P, van de Weerdt BC, Vainio S, Nolan GP, Clevers H. 2001. Wnt signaling is required for thymocyte development and activates Tcf-1 mediated transcription. Eur J Immunol 31:285–293.

Surh CD, Ernst B, Sprent J. 1992. Growth of epithelial cells in the thymic medulla is under the control of mature T cells. J Exp Med 176:611–616.

Tamai K, Semenov M, Kato Y, Spokony R, Liu C, Katsuyama Y, Hess F, Saint-Jeannet JP, He X. 2000. LDL-receptor-related proteins in Wnt signal transduction. Nature 407:530–535.

Ueno T, Saito F, Gray DH, Kuse S, Hieshima K, Nakano H, Kakiuchi T, Lipp M, Boyd RL, Takahama Y. 2004. CCR7 signals are essential for cortex–medulla migration of developing thymocytes. J Exp Med 200:493–505.

Wodarz A, Nusse R. 1998. Mechanisms of Wnt signaling in development. Annu Rev Cell Dev Biol 14:59–88.

